# PROTOCOL: Incentives for climate mitigation in the land use sector: a mixed‐methods systematic review of the effectiveness of payment for environment services (PES) on environmental and socio‐economic outcomes in low‐ and middle‐income countries

**DOI:** 10.1002/CL2.209

**Published:** 2018-05-15

**Authors:** Birte Snilstveit, Jennifer Stevenson, Laurenz Langer, Joshua Polanin, Ian Shemilt, John Eyers, Paul J. Ferraro

## Background

### The problem, condition or issue

Around a quarter of all anthropogenic greenhouse gas emissions originate from the agricultural, forest and other land use sector (AFOLU), driven primarily by deforestation, forest degradation and emissions from unsustainable livestock, soil and nutrient management practices (IPCC, 2014). But there is also a large potential for climate change mitigation in the sector, through removal of greenhouse gases in the atmosphere (carbon sequestration) and reduction in emissions from reduced forest and vegetation removal and improved agricultural practices.

AFOLU sector^1^ also provides a range of other ecosystem services in addition to climate regulation. Forests and lands provide clean water, regulate soil and provide food, fuel, fiber and fresh water (MEA, 2005). Agriculture provides directly and indirectly for the livelihoods of billions of people, in addition to providing food for all the world's population (FAO, 2016a). The sector also offers livelihoods for an estimated 750 million of the world's extreme poor (FAO, ibid). Finally, forests provide paid employment for at least 100 million people and support the livelihoods of many millions more (FAO, 2016b).

The United Nations Framework Convention for Climate Change (UNFCCC) have recognised the critical importance of reducing emissions from deforestation and degradation for climate mitigation (UNFCCC, 2010). In addition, the IPCC highlights the importance of preservation and restoration of other ecosystems such as peatlands and mangroves for maintaining carbon stocks and reducing emissions (FAO & IPCC, 2017; IPCC, 2014). Improved livestock and crop management also represent practices with mitigation potential (FAO & IPCC, ibid).

The links between climate change, agriculture, forests and human wellbeing are complex.

The world's forest area declined from 4128 million hectares of forest in 1990 to 3 999 million hectares in 2015 (FAO, 2016c). Agriculture, both commercial and subsistence, was the main driver of this global deforestation, accounting for 73 per cent of forest clearance worldwide (FAO, 2016b). This is partially driven by an increasing global demand for food from increasing incomes and growing populations, which is expected to rise 60 per cent from 2006 levels by 2050 (FAO, 2016a). At the same time, climate change is expected to negatively affect all dimensions of food security, including agricultural production of food, quality, food access through the impacts on livelihoods, and food price stability (IPCC, 2014).

These complex relationships make sustainable preservation and management of forests and land, while at the same time ensuring food and livelihoods for the world's population, one of the biggest policy challenges facing the world (FAO, 2016a; FAO, 2016b). Concerns that climate change mitigation programming may have negative knock‐on effects on human wellbeing and human rights, especially for the poor, remain. (Stickler 2009; Larson et al. 2013; Lawlor et al. 2013; Mutabazi et al. 2014). It is therefore important to identify strategies that reduce trade‐offs between environmental protection and human wellbeing, and ideally programmes that offer win‐win solutions.

### The intervention

Economic incentives‐based programmes, which aim to preserve or restore ecosystems services through financial incentives, have grown in popularity in the last two decades (Pirard, 2012; GEF, 2014; Ezzine‐de‐Blas et al., 2016). One such incentive‐based mechanism is Payment for Environmental Services (PES). PES are a market‐based approach, where users of an environmental service pay the owners or managers of the service, conditional on changes in behaviours that are likely to effect the provision of environmental services (Wunder, 2015). PES may be conditional on commitments to protect or restore forest areas or sustainable forest management, such as management of forest fires (Jayachandran et al., 2016; Alix‐Garcia et al., 2014). Payments may also be tied to agricultural practices associated with reduction in GHG emissions or increase of carbon stocks, including introduction of agroforestry, silvo‐pastoral or integrated crop systems, which combine crops, grazing lands and trees on agricultural land, improved tillage practices such as conservation agriculture, and reduced use of fire in rangeland management (Hedge & Bull, 2011; Garbach et al. 2012).

There is some debate on the definition of PES (Wunder, 2015; Muriadian et al. 2010; Engels et al. 2008). At the most simple level, PES is a voluntary transaction between service users and service providers, conditional on agreed rules for natural resource management that aims to generate environmental services or benefits that are felt off‐site, for example carbon sequestration (Wunder et al. 2015). In practice, the service “user” is typically a government or NGO acting on behalf of beneficiaries of the environmental service and the service “providers” are individuals, households or community organisations that own or manage the land or forest areas in the programme.

There are a number of long‐standing PES programmes in existence around the world, for example the Pago por Servicios Ambientales‐Hidrologico (PSAH) in Mexico and the Sloping Land Conversion Programme (SLCP) in China. The PSAH in Mexico makes payments to landowners conditional on maintenance of certain level of forest cover, according to five‐year contracts (Alix‐Garcia et al., 2014). If forestland is converted to another land use such as agriculture, the landowner is removed from the programme. The SLCP in China is a large‐scale programme that aims to incentivise the conversion of cropland back to forests or grassland through cash and in‐kind payments to participating households, to reverse or prevent soil erosion and desertification (Démurger & Wan, 2012). In addition to these long‐standing programmes, the number of new PES programmes has grown rapidly in the last decade (Börner et al., 2017). They increasingly also include goals around poverty alleviation.

For example, while the original goal of the PSAH was to maintain the provision of hydrological services from Mexico's forested land, in 2006 the objectives were extended to alleviating poverty (Alix‐Garcia et al., ibid).

Because of the restrictions around land use from participating in the programme, implementers of PES programmes sometimes combine them with other activities to support behaviour change, such as awareness raising activities around environmental conservation or capacity building in sustainable resource use (Sharma & Pattanayak, 2015). In some cases they are also combined with more extensive support for livelihoods development. For example, a REDD+ pilot programme in Nepal made incentive‐based payments to Community Forest User Groups (CFUGs). In addition to forest carbon monitoring, this programme included awareness raising and capacity building for improving local livelihoods and the use of alternative fuel and cooking technologies (Sharma & Pattanayak, ibid).

### How the intervention might work

Payments for Environmental Services (PES) are frequently framed as a response to “market failure” (Arriagada & Perrings, 2009). A market failure occurs when the market does not provide a socially optimum level of a service or good because of the presence of positive externalities for society from providing the service. Carbon sequestration is an example of a public good with positive externalities felt at the global level (Alix‐Garcia & Wolff, 2014). While households may get some individual benefits from environmental practices such as keeping trees on land, the larger benefits are felt externally but households are not compensated financially for these external benefits by market mechanisms. This leads to household or individual decisions that are sub‐optimal for society, like deforestation.

**Figure 1:**
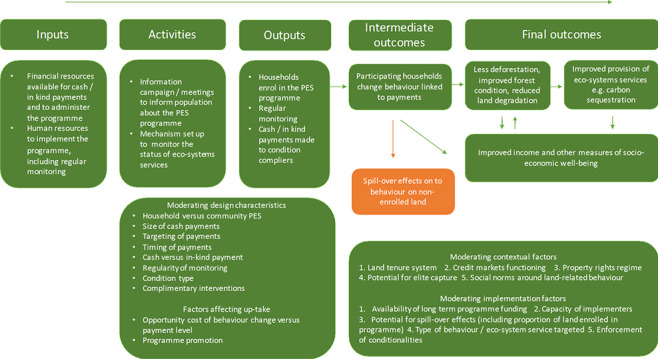
PES programme theory

The overarching theory of how PES works is quite simple. It is designed to act as an incentive for a household or community to contribute to the provision of a socially‐optimal level of environmental services, thus correcting the market failure. Figure 1 presents a programme theory for how PES may influence environmental and socio‐economic outcomes. The outcomes presented in the model are not the only potential outcomes of PES programmes, however we have chosen to focus on those that are of direct interest in this review.

**How PES may influence environmental outcomes**

The intervention aims to influence environmental outcomes primarily through provision of a positive financial incentive to change environment‐related behaviours (Pattanayak et al., 2010). Cash or in‐ kind payments are typically made to participating individuals, households or communities on a regular basis, conditional on the environmental behaviour, for example, payments to landowners to avoid deforestation on their land. Payments may come from private actors that directly benefit from the environmental service, but more typically come from government or non‐governmental organisations acting on their behalf. If a participating household or community organisation fails to uphold the minimum environmental service provision, payments are suspended.

The theory underlying PES is that the financial incentives motivate participants to comply with the rules of the programme, resulting in improved land or forest management practices (Alix‐Garcia & Wolff, 2014). The theory is that the increase in take‐up of these improved practices will ultimately restore, maintain or enhance the provision of the environmental service that has wider benefits for society. The theory assumes that the conservation payments outweigh the benefits derived from business as usual, such as converting forests to agricultural uses, or harvesting wood for energy.

PES may have positive or negative spill‐over effects on land that is not enrolled in the programme. If households or communities do not enrol all their land in a programme, resource exploitation pressures may simply move on to the non‐enrolled areas, known as leakage or substitution effects (Sills et al., 2008). Similarly, increased household income because of the PES programme may have implications for spending patterns and put increased pressure on local resources (Börner et al., 2017). Conversely, positive spill‐overs may occur due to increased forest monitoring resulting from the program or changes in social norms relating to resource use. Such indirect effects can affect the magnitude or even the direction of the effect of a PES programme (Pattanayak et al., 2010).

**How PES may influence environmental and socio‐economic outcomes**

While not originally intended as a tool for poverty alleviation, PES may increase income for complying individuals or households. To directly increase household income, the assumption is that the cash transfer is greater than lost rents previously generated from the enrolled land. Alternatively, payments may also indirectly act as an incentive for households to diversify towards other livelihood activities that are less reliant on practices that reduce the provision of the ecosystems services. For example, participants may move away from agriculture that relies on regular forest clearing towards sustainable forest activities.

However, there are potential trade‐offs between poverty alleviation and environmental goals. The effectiveness of PES in improving environmental outcomes is theorised to depend on effective targeting towards those actors that are the biggest threat to the provision of the environmental service (Wunder, 2007; Börner et al., 2017). If the biggest threat comes from larger, better off households or communities, the payment is best targeted towards them, but this will come at the cost of income transfers to poorer families that could support poverty alleviation (Alix‐Garcia & Wolffe, 2014).

A range of programme design, implementation and contextual factors may influence the effectiveness of PES programmes. Below are some key design, implementation and contextual variables that are frequently theorised to moderate the effectiveness of PES schemes. In many cases, the theory is not conclusive on whether the impact on effectiveness would be positive or negative and thus on the direction of effects of PES schemes in general (Ferraro, 2017; Pattanayak et al., 2010). These factors will be explored in the review in the analysis of heterogeneity.

- *Targeting can influence whether PES programmes achieve their objectives.* PES programmes are typically voluntary and there is a risk that households that already meet conditions will self‐select into the programme. Depending on the opportunity cost of participating in the programme, households may choose to not enrol or only enrol some of their land (Ferraro, 2017). Land enrolled in PES programmes may therefore be land with the lowest value in terms of exploitation potential and thus the least likely to be exploited in the absence of PES. The result of this would be little or no added benefit of the programme in terms of environmental outcomes as land owners may have preserved resources even in the absence of payments.
  The lack of additionality may therefore be more prevalent where pre‐programme compliance with PES conditions is high (eg: low levels of resource exploitation, as indicated by low baseline deforestation rates for example). Thus, programmes targeted to land that is at a high risk of exploitation may result in higher levels of resource protection. However, this involves predicting where landholders will exploit resources in the future, information that is generally hidden from the policy‐maker implementing the PES programmes (Alix‐Garcia & Wolffe, 2014).
- *The size of payments may influence take‐up and the extent to which programme participants change their behaviour:* If the cost of lost rents from restrictions on land or resource use from participating in the programme are greater than the payments received, a land owner is unlikely to choose to enrol. This requires a payment that is large enough to overcome the opportunity costs for households to decide to participate in the programme and then to stick to conditions. However, because of missing markets the payment size that will induce people to participate in the programme is not observed (Börner et al. 2017).
- *Timing of payments:* the timing of payments may influence how programme participants respond to the financial incentive. There is some suggestion that payments made at the end of the contracted period are most effective at incentivising changes in environmental behaviours (Alix‐Garcia & Wolffe, 2014). However, this is often not feasible, particularly in low‐income contexts, and often payments are made on a yearly basis.
- *The characteristics of PES conditions:* Even if an improvement in an ecosystem service is the goal of a programme, few PES programmes are conditional on the provision of the ecosystem service itself (such as demonstrated increases in carbon sequestration in forests). In practice, PES program payments are frequently conditional on proxies or changes in behaviours that are likely to affect the provision of the ecosystem service (Wunder, 2015). For example, planting trees on agricultural land to improve carbon sequestration. While the use of proxies is typically easier to observe, there is no guarantee that changes in the behaviour will lead to improved ecosystems provision, particularly where the ecosystem service is heavily influenced by external factors to the programme (Pattanayak et al., 2010; Borner et al. 2017).
- *The extent to which conditions are monitored and enforced* may moderate effects on environmental outcomes (Börner et al. 2017). Monitoring and enforcement of conditions may influence the extent to which programme participants change their behaviour and comply with conditions. A systematic review of the effects of cash transfer payments for building human capital found a larger effect on children's education outcomes when conditions were monitored and enforced (Baird et al., 2013).
- *Long run programme funding:* permanent benefits of PES schemes may depend on continuous programme funding, which may be particularly difficult in government run PES schemes (Engel et al., 2008). On the other hand, payments may act to incentivise people to incur the fixed costs of switching to a more environmentally friendly practice and to “learn by doing” (learn about benefits and learn to reduce variable costs). And once they adopt a new practice, the marginal benefits may outweigh the marginal costs and the practice will persist even in the absence of payments.
- *Property rights system:* weak property rights are a common driver of deforestation and lack of secure property rights may make PES implementation difficult (Alix‐Garcia & Wolffe, 2014). Lack of secure property rights may reduce programme take‐up rates and compliance as participants are less willing to invest in the sustainable management of land when they are uncertain if they will be able to reap benefits from those investments in the future.
- *Land tenure system*: incentives to change behaviour around land management practices may depend on whether the land is privately owned, collectively owned, state owned or restricted in some way by the state (Robinson et al., 2017). For example, PES payments may have weaker effects on conservation behavior of users living in or near state owned lands than on private land or land held under collective title.
- *Credit markets:* the presence of credit constraints for poor families in LMICs may be a barrier for them to make investments in, or exploit, land (Ferraro, 2017). There may be negative environmental consequences when payments to participating families allow them to overcome these constraints to make investments in unenrolled land, or enrolled land once payments stop, that result in less environmentally favourable land uses.

### Why it is important to do the review

***Review of existing literature***

There is an emerging impact evaluation literature on payments for environmental services (PES) programmes. A 3ie evidence gap map (EGM) published in 2016 identified 41^2^ experimental or quasi‐experimental evaluations of PES programmes globally, with most taking place in Low‐and Middle‐Income Countries (L&MICs). We are only aware of one systematic review on the effectiveness of PES, published in 2014 (Samii et al., 2014). There have also been a large number of non‐systematic literature reviews, either presenting narrative discussions on the effectiveness of PES (Börner et al., 2017; Pattanayak et al., 2010; Alix‐Garcia & Wolffe, 2014) or presenting a range of effect sizes for PES programmes (Ferraro, 2017).

There are several reasons that warrant an update and extension of the Samii et al. (2014) systematic review. Firstly, the search for the review was completed in August 2013. 3ie's Evidence Gap Map of land use and forestry programmes (Snilstveit et al., 2016) identified at least six new evaluations of PES programmes that have been published since then, including studies from Uganda, Ecuador, Tanzania and new evaluations of long‐term programmes in China, Mexico and Costa Rica. Secondly, Samii et al. (2014) were unable to do a meta‐analysis for income and poverty related outcomes and for forest condition due to lack of data and heterogeneity between studies. Given the increase in the evaluation evidence base since then, we hope to be able to undertake additional meta‐analyses.

Thirdly, Samii et al.'s review focused on PES for forest areas. We will expand the scope of the review to include PES in other settings such as farmland, mangroves and grasslands. A number of PES programmes target other important environmental behaviours of relevance to climate change mitigation programming, for example payments to incentivise farmers to take up agroforestry on their farmland (Hedge & Bull, 2011). This will be the first review that we are aware of to systematically cover the literature on the effectiveness of PES in these areas.

Finally, this review will answer new questions around design, implementation, context and costs of programmes, in addition to assessing programme effects. In doing so we will look at a broader range of literature, including process evaluations, programme documents and associated qualitative studies for the programmes evaluated in included impact evaluations.

*Relevance to policy and practice*

It is estimated that additional global investments of US$35 billion in the agriculture sector and US$21 billion in the forestry sector will be needed by 2030 to mitigate the effects of climate change (UNFCCC 2009). At the landmark United Nations Climate Change Conference (COP 21) in 2015, countries agreed to conserve and enhance sinks of greenhouse gases, including forests (UNFCCC, 2015). To ensure resources are used effectively to achieve agreed mitigation objectives it is important to ensure that decision‐makers have access to reliable evidence.

The United Nations Reducing Emissions from Deforestation and Forest Degradation mechanism (REDD+) is one of the main frameworks for making payments to L&MICs to preserve and sustainably manage forests. There are significant resources pledged to the REDD+ initiative. At the COP21, Germany, Norway and the UK announced that they would provide US$ 5 billion between 2015 and 2020 to forest countries if they could demonstrate verified emissions reductions (BMUB, 2015). The UN‐REDD Programme currently supports 64 countries across Africa, South and East Asia and Latin America and the Caribbean to enable their participation in REDD+, and 47 so far have qualified (UN‐REDD, 2016).

PES are promoted as an important tool by REDD+ and are supported by a range of actors, from national governments to multi‐national institutions such as IFAD, UNDP and the World Bank (GEF, 2014). The number of PES programmes operating in L&MICs has rapidly increased. A recent global review of PES identified hundreds of programmes mentioned in the literature, with 55 programmes currently in operation around the world that clearly fit the classic definition of PES (Ezzine‐Blas et al., 2016). The Global Environmental Facility (GEF) alone has supported 57 projects containing elements of PES since its inception, totalling investments of over $225 million, in addition to $1.59 billion leveraged from co‐financing (GEF, 2014).

Despite their popularity, key policy questions around the effectiveness of PES remain unanswered (Samii et al., 2014; Ferraro, 2017; Le Velly & Dutilly, 2016). One of these questions is the extent to which the environmental and poverty reduction goals of such a programme conflict or present strategies that can generate both environmental and poverty reduction benefits. A second, and equally important question is if PES generate environmental benefits that are additional to ‘business as usual’. To meet UNFCCC emissions targets, governments implement PES programmes on the assumption that by compensating some groups to reduce their emissions, emissions in other sectors are offset (Nhantumbo & Camargo, 2015).

Evaluations of PES programmes finding small effects have led some to dismiss it as an important mechanism. Indeed, a recent FAO‐IPCC (2017) report on climate change and land use following the Paris Agreements stated that “*[PES] effectiveness, however, is limited and they are more readily applied in some sectors (e.g. forest management) than in other emerging concerns (land restoration, soil health and soil carbon*)” (FAO‐IPCC, 2017: 28). The report concludes that for PES programmes to be effective, they must be better designed and informed by meta‐analysis of the effects of previous programmes.

A range of policy alternatives to PES exist, including private sector zero‐deforestation commitments (Climate Focus, 2015) and community forestry initiatives (Agrawal & Angelsen, 2009; Angelsen, 2009). The effectiveness of many of these approaches is also contested and should be subject of future reviews. While PES may be one of the most popular policy tools in the sector, it is important to assess the relative costs and effectiveness of the approach, facilitating comparison with other options in the future.

Given the resources dedicated to PES and the global importance of effective climate change mitigation activities, it is essential that rigorous and comprehensive evidence is available to policy‐makers and implementers. To help inform decisions about how to use available resources most effectively we will provide a comprehensive review and synthesis of the evidence on the effects of PES, including an assessment of how intervention design, implementation and contextual factors moderate outcomes and cost‐effectiveness.

## Objectives

The objective of this review is to assess the effects of PES programmes on environmental and socio‐economic outcomes in low‐ and middle‐income countries (L&MICs). This will include identifying and synthesising evidence on how PES programme effects vary by programme design, implementation, context^3^; and by sub‐groups of PES programme participants. We will also attempt to assess the cost‐effectiveness of PES programmes.

To address these objectives, we will answer the following questions:

1) What is the effectiveness of PES programmes on intermediate, environmental and socio‐economic outcomes in L&MICs?
  a) Do PES programs simultaneously deliver positive environmental and socio‐economic effects?
  b) Do effects vary by sub‐groups of people participating in PES programmes, including low‐income groups, women and indigenous people?
  c) Do effects vary by type of environmental services targeted?
2) To what extent do design and implementation features moderate the effectiveness of PES programmes?
3) In which contexts are PES programmes effective (or ineffective)? What are the contextual barriers to, and facilitators of, programme effectiveness?
4) What is the cost‐effectiveness of PES programmes?

## Methodology

The review will follow the Campbell and Cochrane Collaborations' guidelines to systematic reviewing (The Steering Group of the Campbell Collaboration, 2016; Hammerstrøm et al., 2010; Higgins & Green, 2011; Shadish & Myers, 2004). The review will also draw on the concepts of theory‐based impact evaluation (White, 2009) and theory‐based systematic reviews (Snilstveit, 2012; Waddington et al., 2012) to provide a mixed‐methods systematic review and analysis along the causal chain, to also address questions related to intervention design, implementation and context.

To do so we will include studies in two phases. To address questions 1a, b and c, we will include studies meeting the impact evaluations study design criteria, presented below. To address questions 2, 3 and 4, studies that meet these criteria will be used as the basis for a second, targeted search to identify and include qualitative studies, project documents, process evaluations and cost data on the programmes examined.

### Criteria for including and excluding studies

***Types of population***

We will include studies of programmes in countries classified by the World Bank as lower income, lower‐middle income, or upper middle income (L&MICs). We use the classification of the country in the year of the initiation of the program under study. There are several reasons why we decided to focus on L&MICs only. Some scoping of the literature suggests that the impact evaluation literature on PES from high‐income countries (HICs) is significantly smaller and does not typically use methods that would be included in the review (Snilstveit et al., 2016; Schomers & Matzdorf, 2013). It does not typically self‐identify as PES (Schomers & Matzdorf, 2013; Ezzine‐de‐Blas et al., 2016) and would likely result in a need to search a separate literature. This is likely to add a significant amount of work to the searching and screening with only a potentially very small number of included studies. In addition, L&MICs contain most of the world's tropical forests, which offer the greatest potential for climate change mitigation in the AFOLU sector, such as climate regulation, watershed protection and carbon sequestration (Pattanayak et al., 2010). Similarly, the findings from the HIC literature will be less relevant for mechanisms such as REDD+. Finally, given that one of our main objectives is understanding the potential for PES to offer “win‐win” environmental and poverty alleviation solutions, L&MIC settings offer a more likely setting for answering this.

We will include studies targeted at populations living in or near to forests, agricultural land, wetlands, grasslands and mangroves. Forests are defined as an area over 0.5 hectares with trees higher than five metres and canopy cover more than 10 per cent (FAO, 2012), including mangrove forest areas. Grasslands are areas with tree or shrub canopy cover below 10 per cent but with herbaceous plant cover (FAO, 2005).

Studies of programmes in HICs will be excluded.

***Types of interventions***

We will include studies of PES programmes, defined as those providing payments to owners or managers of land, conditional on some minimum environmental/ ecosystems service provision. Payments can be either cash or in‐kind material transfers, such as seedlings, api‐culture and fencing. Ecosystems services are defined as the benefits that humans get from ecosystems (MEA, 2005). In ideal type PES programmes, payments are conditional on the provision of the ecosystem service itself, for example payments for increased carbon sequestration in forests (Le Velly & Dutilly, 2016). However, in practice most PES program payments are conditional on changes in behaviours that are likely to affect the provision of the ecosystem service, for example reducing deforestation or planting trees on agricultural land. We will include payments tied either to the provision of an ecosystem service or to any of the following practices related to climate‐regulating ecosystems services: forest protection or regeneration; sustainable forest management practices; sustainable watershed management; sustainable agricultural practices; sustainable livestock management.

The payments can be made to an individual, household, community or organisation and can either be conditional on a specified environmental commitment, for example on the fulfilment of an obligation to maintain a certain forest cover on land, or paid in advance of the PES programme. We will not limit inclusion of these programmes by the funder/ implementer (private versus public for example) or status of land (private land or state‐owned/ protected land). Finally, we will include programmes that study PES alone or in combination with other intervention activities, for example interventions supporting alternative livelihoods.^4^

***Types of outcomes***

We will include studies that assess the impact of PES on either environmental, socio‐economic or intermediate outcomes, as defined below. PES programmes often have multiple objectives, related to both the preservation or restoration of environmental services and human welfare. There is a considerable literature on the potential trade‐offs or complementarities between these objectives. By looking at both sets of outcomes, we aim to inform this debate.

We will also include studies that assess intermediate outcomes such as changes in agricultural, forest or land management practices. This will allow us to report on effects at earlier stages of the PES causal chain.

***Intermediate outcomes***

We will include studies that assess changes in land or forest management practices, defined as measures of the type, frequency, intensity or adoption of such practices at the household or community level. We will also include studies that assess the adoption of sustainable agricultural practices or technologies, for example incorporating trees into agricultural or grazing lands. We will also assess measures of forest dependence, for example resource extraction.

***Environmental outcomes***

We will include environmental outcomes that are related to greenhouse gas emissions or carbon storage/ sequestration. This includes both direct measures of emissions (CO_2_, CH_4_, N_2_0) or carbon storage/ sequestration and proxies for such outcomes. Based on previous mapping work in this area, we know that there are few evaluations that measure provision of environmental services such as carbon sequestration (Snilstveit et al. 2016). Proxy outcomes include deforestation rate, forest cover, forest condition/ degradation, forest fires, soil quality, and so on. We will accept whichever measure is used by the study authors. Once we have identified all studies, we will map all outcomes to determine if they are sufficiently similar for meta‐analysis.

We will also include outcomes related to the spillover effects of PES programmes on to land or forests not enrolled in PES programmes.

***Socio‐economic outcomes***

We will include any measures of socio‐economic outcomes, including income, consumption, well‐being, livelihood security and assets of communities / households / individuals participating in PES programmes. We will also include measures of food security across the four dimensions of food availability, access, utilisation and stability included in the Declaration on Food Security (FAO 2009). These include food consumption, food expenditure, prevalence of undernourishment and nutritional status (FAO 2013). We will accept whichever socio‐economic measure is used by the study authors. Once we have identified all studies, we will map all outcomes to determine if they are sufficiently similar for meta‐analysis.

### Types of study designs

We will include studies in two stages, in a similar approach to Snilstveit et al. (2015). In the first stage, we will include studies that assessed the effects of interventions using experimental designs or quasi‐experimental designs with non‐random assignment that allow for causal inference (to address primary research question 1). Specifically we will include the following:

- Studies where participants are randomly assigned to treatment and comparison group (experimental study designs);
- Studies where assignment to treatment and comparison group is based on other known allocation rules, including a threshold on a continuous variable (regression discontinuity designs) or exogenous geographical variation in the treatment allocation (natural experiments);
- Studies with non‐random assignment to treatment and comparison group that include pre‐and post‐test measures of the outcome variables of interest to ensure equity between groups on the baseline measure, and that use appropriate methods to control for selection bias and confounding. Such methods include statistical matching (for example, propensity score matching, or covariate matching), regression adjustment (for example, difference‐in‐differences, fixed effects regression, single difference regression analysis, instrumental variables, and ‘Heckman’ selection models).
- Studies with non‐random assignment to treatment and comparison group that include post‐test measures of the outcome variables of interest only and use appropriate methods to control for selection bias and confounding, as above. This includes pipeline and cohort studies.

Ferraro and Miranda (2014; 2017) argue that combining panel data with baseline observations and statistical matching is the most effective quasi‐experimental method at reducing bias when evaluating conservation sector programmes. However, given the expected small size of the evidence base, we will include studies with post‐intervention outcome data only as long as they use some method to control for selection bias and confounding. To account for the differences in the quality of study designs and analysis methods, we will appraise the risk of bias in all included studies and do sub‐group analysis by risk of bias status.

Before‐after studies and observational studies without control for selection bias and confounding will be excluded. Additionally, modelling based studies, commentaries and literature reviews will be excluded.

To address questions 2 and 3 on programme design, implementation and context, we will extract descriptive and qualitative data from the included experimental and quasi‐experimental studies. In addition, we will conduct a targeted search for additional papers on the programmes covered by the included impact evaluations to provide additional detail on these areas. In order to be included, these papers must be related to the programmes in the included impact evaluations and also be one or more of the following types of studies^5^:

- A qualitative study collecting primary data using qualitative or quantitative methods of data collection and analysis, and reporting some information on all of the following: the research question, procedures for collecting data, sampling and recruitment, and at least two sample characteristics.
- A descriptive quantitative study collecting primary data using quantitative methods of data collection and descriptive quantitative analysis and report some information on all of the following: the research question, procedures for collecting data, sampling and recruitment, and at least two sample characteristics
- A process evaluation assessing whether a programme is being implemented as intended and what is felt to be working more or less well, and why (HM Treasury, 2011). Process evaluations may include the collection of qualitative and quantitative data from different stakeholders to cover subjective issues, such as perceptions of intervention success or more objective issues, such as how an intervention was operationalised. They might also be used to collect organisational information;
- A project document providing information about planned, ongoing or completed programmes. They may describe the background and design of an intervention, or the resources available for a project for instance. As such, these documents do not typically include much analysis of primary evidence, but they provide factual information about interventions. The purpose of including them in our review is to ensure we had sufficient information about the context and interventions in included studies

To address question 4 on cost‐effectiveness we will include economic evaluations. We will also use any economic evaluation or cost data provided in any of the studies included under the criteria above.

***Types of comparison***

We will include studies with a comparison group that receives no intervention (including wait‐list comparisons), business as usual, or a different environmental intervention.

Studies that only include a temporal (before‐after) comparison will be excluded.

***Other criteria for including and excluding studies***

We will not impose any restriction on inclusion of studies by language of publication or publication status. However, we will undertake our searches in English. We will search the literature back to 1990, excluding any studies published before this date. This date date cut off is justified by both previous reviews of the literature, as well as the implementation of PES as a policy instrument for reducing deforestation. An evidence gap map covering PES interventions that searched back to 1990 did not identify any studies published before 2000 (Puri et al., 2016). Moreover, PES was pioneered by Costa Rica as an approach to reducing deforestation in the late 1990s (add ref) and REDD was first discussed at the UNFCCC conference of the parties in 2005 (UNFCCC, 2005). Thus implementation and studies of PES is unlikely to have taken place before 1990.

An overview of the inclusion criteria is provided in Table 1.

**Table 1:** Summary of inclusion criteria

**Characteristics**	**Inclusion criteria**
**Population**	Populations living in or near forests, wetlands, grasslands, mangroves and farmland areas in countries classified by the World Bank as Low‐or‐Middle Income (LMICs)
**Interventions**	Payments for environmental services programmes
**Comparisons**	Comparison group that receives no intervention (including wait‐list comparisons), business as usual, or a different environmental intervention
**Outcomes**	Intermediate, environmental and socio‐economic outcomes
**Study design**	To answer question 1, experimental and quasi‐experimental studies. To answer questions 2 and 3, qualitative studies, descriptive quantitative studies, process evaluations, project documents
**Other**	No inclusion restrictions by publication status or language.

### Search strategy: Studies to address review question 1

We will implement a systematic and comprehensive search strategy, developed in consultation with an information specialist, as outlined below.

**Electronic searches**

We will search a range of databases and websites, including general sources of social science literature as well as sources specific to climate change, forestry, agriculture and impact evaluation. To reduce the potential for publication bias, this will include both academic databases as well a range of specialist organisational websites and repositories of impact evaluations in international development. The sources covered by the search are listed below.

Bibliographic databases:

- CAB Abstracts: http://www.cabi.org/publishing‐products/online‐information‐resources/cab‐abstracts/
- Web of Science: http://wok.mimas.ac.uk/
- Greenfile (EBSCO): https://www.ebscohost.com/academic/greenfile
- Econlit: https://www.aeaweb.org/econlit/
- AgEcon: https://ageconsearch.tind.io/?ln=en
- IDEAS/RePeC (EBSCO Discovery): https://www.ebscohost.com/discovery
- Agris (EBSCO Discovery): https://www.ebscohost.com/discovery

Specialist organisational databases:

- Centre for International Forestry Research (CIFOR): http://www.cifor.org/library/
- International Food Policy Research Institute Library (IFPRI): http://library.ifpri.info/discover/collections/
- International Institute for Environment and Development (IIED): http://pubs.iied.org/about/
- World Agrofresty Centre (ICRAF): outputs.worldagroforestry.org/
- ATAI Research: https://www.atai‐research.org/emerging‐insights/?
- Global Environment Facility Evaluation Office: http://www.gefieo.org/evaluations/all?f[0]=field_ieo_grouping%3A312
- Conservation Evidence: http://www.conservationevidence.com/
- Climate Change Agriculture and Food Security (CCAFS) publications: https://ccafs.cgiar.org/publications
- Conservation International publications: http://www.conservation.org/publications/Pages/default.aspx
- IUCN Library: https://portals.iucn.org/library/dir/publications‐list
- Biodiversity International: http://www.bioversityinternational.org/e‐library/publications/

Bilateral and multilateral agencies and general repositories of impact evaluations in international development:

- World Bank Open Knowledge Repository: https://openknowledge.worldbank.org/
- DFID Research for Development (R4D): http://r4d.dfid.gov.uk/
- Inter‐American Development Bank Publications: https://publications.iadb.org/facet‐view?locale‐attribute=en&field=type_view
- African Development Bank (AfDB): https://www.afdb.org/en/documents/publications/
- Asian Development Bank (ADB): https://www.adb.org/publications
- United Nations Development Programme (UNDP): http://www.undp.org/content/undp/en/home/library.html
- United National Environmental Programme: http://www.unep.org/publications/
- International Fund for Agricultural Development (IFAD): https://www.ifad.org/pub/overview
- Food and Agriculture Organisation of the United Nations (FAO): http://www.fao.org/publications/en/
- 3ie Repository of Impact Evaluations http://www.3ieimpact.org/en/evidence/impact‐evaluations/
- 3ie RIDIE (Registry for International Development Impact Evaluations): http://ridie.3ieimpact.org/
- Innovations for Poverty Action (IPA): http://www.poverty‐action.org/projectevaluations

J‐Poverty Action Lab: https://www.povertyactionlab.org/evaluations

**Other searches**

We will screen the bibliography of existing systematic reviews, literature reviews and evidence gap maps for eligible studies, including the systematic review that this review will update and extend (Samii et al., 2014), and recent evidence gap maps (Snilstveit et al., 2016; Puri et al., 2016). We will also screen the reference lists of included studies and undertake forward citation‐tracking for those studies using Google Scholar.

We will contact key experts and organisations working in the sector to identify additional studies.

### Targeted search for addressing review questions 2 and 3

Once we have identified our set of included impact evaluations, we will undertake targeted searching for qualitative studies, process evaluation, project documents and economic evaluations for those interventions evaluated in the included studies. We will conduct citation tracking of included studies to identify relevant sister papers and conduct internet and database searches using the names of programs from included studies. To identify project documents and process evaluations we will conduct targeted searches of databases of project documents and websites of implementing agencies. Finally, we will contact authors and implementing agencies to request available project documentation.

**Screening**

We will import all search results into EPPI‐Reviewer 4. Once duplicates have been removed we will screen citations against review inclusion criteria at title/ abstract and full‐text. At the title/abstract screening stage, we will make use of innovative text mining technologies to speed up the initial screening workload and test the potential for reductions in screening workload (O'Mara‐Eves et al., 2015; Shemilt et al., 2016). We will use two functions in EPPI Reviewer to do this: the priority‐screening function and inclusion/ exclusion classifier. We will rely on the first option in the list below to include studies in the review, but will compare the results of 2 and 3 retrospectively to assess reliability:

1. Full independent double screening using the priority screening function to order results by probability of inclusion, based on a training set of screening;
2. Single screening using the priority screening function with a “safety first” approach (an option to mark unclear studies for review by a second screener) (Shemilt et al., 2016);
3. Single screening using the priority screening function combined with the use of the classifier function to auto‐exclude studies with a very low probability of inclusion;

The priority screening function can be used at the title/ abstract screening stage to prioritise the items most likely to be ‘includes’ based on previously included documents. This will involve screening a random test set of at least 500 citations to train the priority screening function, which will learn to identify relevant records based on key‐words in the title and abstract of the included and excluded studies. Using priority screening in this way allows for the identification of includable records at an earlier stage in the review process so that work can begin earlier on full‐text screening and data extraction. We will do this using both independent double screening. We will also have a single reviewer doing the screening independently with a safety first approach in order to compare results. We will also use the priority screening function to develop a classifier that will retrospectively classify studies into groups based on their probability of inclusion in the review. We will test the reliability of automatically excluding studies with a low probability of inclusion (for example less than a 10 per cent chance of inclusion), by comparing the results to the first approach.

Independent double screening is typically considered the most reliable approach to screening in systematic reviews. However, this approach is also very resource intensive. In the ‘single screening with text mining’ approach the machine effectively plays the role of the second screener. Moreover, before applying text mining all reviewers will be allocated the same set of 100 randomly selected records for independent screening to establish inter‐rater reliability, followed by a meeting to discuss any disagreements.

At the full‐text screening stage, a random sample of 20 per cent of the records will be independently double screened by two reviewers, followed by a meeting to discuss any disagreements, to establish inter‐rater reliability in the application of inclusion criteria. For the remaining records, we will move to single screening using a ‘safety first’ approach (Shemilt et al., 2016), with an option for reviewers to put any papers where inclusion is unclear in a ‘provisionally include’ folder for screening by a senior reviewer.

### Data extraction and coding procedures

We will use a standardised data extraction form to extract data from included papers (the full data extraction form is included in appendix 2). We will use Excel and EPPI reviewer and extract data on the following categories of information:

1. descriptive data on study design, intervention and context for purposes of descriptive analysis of the body of research;
2. data on the population, context, study design, intervention design, process and implementation and cost for purposes of moderator analysis and qualitative synthesis addressing questions 2 and 3
3. data on the outcomes of interest and sample size for purposes of effect size calculation

### Critical appraisal

**Assessment of risk of bias in experimental and quasi‐experimental studies**

We will assess the risk of bias in the included impact evaluations using criteria as suggested by an adapted version of the Cochrane Risk of Bias Tool (Hombrados and Waddington, 2012). We will assess risk of bias based on the following criteria, coding each paper as ‘Yes’, ‘No’ and ‘Unclear’ according to how well they address each domain:

- Baseline confounding and selection bias: was the allocation or identification mechanism able to control for baseline confounding and sample selection bias?
- Time‐varying confounding: was the method of analysis executed adequately to ensure comparability of groups throughout the study?
- Bias due to missing data: is the estimation method sensitive to non‐random attrition?
- Biases in outcome data collection: was the process of being observed causing motivation bias (Hawthorne and John Henry effects, courtesy bias, and recall bias)?
- Departures from intended interventions: was the study adequately protected against performance bias and survey effects?
- Outcome & analysis reporting biases: was the study free from outcome reporting bias and analysis reporting bias?

Two reviewers will undertake the risk of bias assessment independently, with disagreements resolved by a third reviewer. We will report the results of the assessment for each of the assessed criteria for each study. We aim to explore if there are systematic differences between primary studies with different risk of bias. If meta‐analysis is feasible, we will conduct sensitivity analysis to assess the robustness of the results to the risk of bias in included studies.

**Assessment of quality in descriptive quantitative studies, qualitative studies and process evaluations**

We will assess the quality of included qualitative studies, process evaluations and descriptive quantitative studies using an adapted version of the Critical Appraisal Skills Programme checklist (CASP, 2006) and Pluye and colleagues' (2011) mixed‐methods appraisal tool. The developed tool will make judgments on the adequacy of reporting, data collection, presentation, analysis and conclusions drawn. The appraisal will assess the quality of the included qualitative studies and descriptive quantitative studies using six appraisal domains:

1. The defensibility of the applied research design to answer the research question under investigation.
2. The defensibility of the selected research sample and the process of selecting research participants.
3. The rigour of the technical research conduct, including the transparency of reporting.
4. The rigour of the applied analysis and credibility of study's claims given the nature of the presented data.
5. The consideration of the study's context (for qualitative studies only).
6. The reflexivity of the reported research.

We will filter out studies of particularly low quality at this stage, using a fatal flaw approach following Dixon‐Woods et (2005). Studies that do not meet either criterion of appraisal domains 1–4 above will be excluded from the synthesis. That is, they will be included in the review and we will report on the studies' descriptive data, for example applied intervention. However, no research findings will be extracted from these studies to feed into the review's synthesis. Each appraisal domain will be assessed from a scale of low quality to medium and high quality. An overall critical appraisal judgement per study will be allocated using a numerical threshold of the appraised quality domains (Appendix XX).

We will not undertake a critical appraisal of included project documents. They typically provide information about planned, ongoing or completed programmes, providing information about the design or resources available for a project for instance. As such these documents do not typically include much analysis of primary evidence, but they provide factual information about interventions. The purpose of including them in our review is to ensure we have sufficient information about the context and interventions included in our review. We will therefore focus the appraisal on assessing the relevance of the documents against the interventions assessed in our review. Before extracting any data, we will ensure that the name of the intervention, the implementing agency, context and timeline of the intervention described in the project document corresponds to the intervention assessed in the impact evaluation included in our review. Finally, collecting data from a range of sources, especially if used for triangulation, can enhance confidence in the trustworthiness of the information included (Montgomery et al., forthcoming). If several sources are available, we will extract data from all sources for purposes of triangulation. If we are doubt about the relevance of a particular document, we will contact the authors.

### Effect size calculation

Where possible we will extract the necessary data to calculate standardised effect sizes. We expect most studies to be measuring continuous outcomes. For these outcomes we will calculate the Hedges' g sample‐size corrected standardised mean difference (SMDs), its variance and standard error using formulae provided in Borenstein et al. (2009, Chapter 4).

The decision as to which formula we use to calculate effect sizes will be made taking into account what has been reported in the majority of the studies sharing common outcomes. We will use the most appropriate formulae for calculating effects sizes, considering the types of study designs we identify and the data they report. Based on our mapping of the literature we expect the majority of included studies to be quasi‐experimental designs with outcome measures reported either as regression coefficients (partial (adjusted) estimates) or mean differences, with standard errors or t‐statistics and sample sizes. Typically studies do not report standard deviations.

We therefore anticipate using one of the formulae listed below (in hierarchical order of preference) (Lipsey & Wilson, 2001):

For studies reporting regression coefficients:

$$d=\frac{2t}{\sqrt{n_{t}+n_{c}}}\;Var_{d}=\frac{2}{n_{t}+n_{c}}+\frac{2}{4(n_{t}+n_{c})}$$

Where n denotes the sample size of treatment group (t) and control (c). We will calculate the t‐statistic (t) by dividing the coefficient by the standard error. If the authors only report confidence intervals and no standard error we will calculate the standard error from the confidence intervals. If the study does not report the standard error, but report t we will extract and use this as reported by the authors.

Studies reporting other data than coefficients and standard errors:

Studies reporting mean differences $\Delta \bar{X}$ (between treatment (T) and control (C) and standard deviation (SD) at follow up (p+1) :

$$d=\frac{\Delta {\bar{X}}_{p+1}}{SD_{p+1}}=\frac{{\bar{X}}_{Tp+1}-{\bar{X}}_{Cp+1}}{SD_{p+1}}$$

Studies reporting mean differences between treatment and control, standard error (SE) and sample size (n):

$$d=\frac{\Delta {\bar{X}}_{p+1}}{\text{SE}\sqrt{n}}$$

Studies reporting means and standard deviations for treatment and control groups at baseline (p) and follow up:

$d=\frac{\Delta {\bar{X}}_{p}-\Delta {\bar{X}}_{p+1}}{SD_{p+1}}$, where

$$\begin{array}{l} SD_{p+1}=\sqrt{\frac{(n_{Tp+1}-1)SD_{T_{p+1}}^{2}+(n_{Cp+1}-1)SD_{T_{p+1}}^{2}}{n_{Tp+1}+n_{Cp+1}-2}}\\ Var_{d}=((\frac{nT+nC)}{nT*nC})+(\frac{d^{2}}{2(nT+nC})) \end{array}$$

Studies reporting proportions (r) in treatment group and control:

$$d=\ln\left[\frac{r_{T}(1-r_{T})}{r_{C}(1-r_{C})}\right]\;\frac{\sqrt{3}}{\pi }$$

Dependent effect sizes can arise when one study provides multiple results for the same outcome of interest or multiple studies use the same dataset and report on the same outcome. Dependent effect sizes are problematic because the traditional estimation of a mean effect size relied on the statistical assumption of independence of each included estimation of effect (Gleser & Olkin, 2007). We expect a large number of PES evaluations will report multiple, dependent effect sizes and therefore this is an important issue to address (Snilstveit et al. 2016). We will therefore follow the rules laid out below for deciding on inclusion in meta‐analysis.

We will only include one effect estimate per sample in a single meta‐analysis. This is with the exception of cases where we identify ten or more effect sizes for the same meta‐analysis; in these cases, we will combine dependent effect sizes within the same meta‐analysis and use robust variance estimation (Hedges, Tipton, & Johnson, 2010; Tanner‐Smith & Tipton, 2014).

Where we identify several papers that report on the same study we will use effect sizes from the most recent publication. Where several studies exist using the same data set or where multiple outcomes are reported from alternate specifications within the same study, we will select the study or specification which is the most similar to other estimates for the same outcome type to enhance the potential for meta‐analysis. Where different studies report on the same programme, but use different samples (for example from different regions) we will include both estimates, treating them as independent samples.

Studies may provide estimates at several different time points. In such cases we will identify the most common follow‐up period and include the follow up measures that match this most closely in the meta‐analysis. Nevertheless we will extract data and calculate effect sizes for all time points and report these in the review.

If we identify studies with multiple treatment arms and only one comparison group, we will estimate a treatment effect from both arms. Ideally, we will code and synthesize both effects within the same meta‐analytic model, accounting for the dependency using robust variance estimation. However, should the meta‐analytic model have less than 10 studies, we will choose the effect estimate from the treatment arm that tests an intervention that most commonly resembles the other interventions included in the meta‐analysis.

**Unit of analysis**

We will assess studies for unit of analysis errors, where the unit of the treatment is different to the unit of analysis, without taking account of clustering in the analysis (The Campbell Collaboration, 2014). If unit of analysis errors exist we will correct for this by adjusting the standard errors using formula provided in Hedges (2011).

**Missing or incomplete data**

We will contact study authors when there is missing or incomplete data for calculating effect sizes. If we are unable to obtain the necessary data, we will report on the descriptive characteristics of the study but state that it was excluded from the meta‐analysis or reporting of effect sizes due to missing data.

### Calculating cost estimates

We will estimate the incremental costs by building a profile of inputs, resource use and costs for each included intervention, drawing on the Ingredients Method (McEwans et al. 2012, Dhaliwal et al. 2012) and the resource‐use data‐coding tool proposed by Shemilt and colleagues (2012). The specification of key intervention “ingredients” will be based on the description of the intervention and the programme theory.

We will use key categories such as personnel, equipment, cash payments, overheads and other programme inputs. We will then capture the quantities of ingredients used, dividing these into fixed and variable costs, and value each input in monetary terms. Where costs of inputs are not available, we will estimate the costs drawing on overall budgets, or using comparable interventions in similar settings.

We will extract data on costs from the included impact evaluations and a range of additional sources including sister papers, as well process evaluations, economic evaluations and programme documents identified through the targeted searches. We will also contact the authors in an attempt to retrieve primary data that can help calculate or estimate intervention costs.

To ensure comparability of cost‐estimates across studies, we will adjust costs for price inflation and currency exchange rates, converting all estimates into the same base year. When costs are provided/e in local currencies in nominal terms, we will convert these into US dollars. All cost conversions will be done using the CCEMG‐EPPI‐Centre Cost Converter (version 1.5, 2016)

### Methods of synthesis

**Review questions 1, 2 and 3: statistical meta‐analysis and meta‐regression**

We will synthesise evidence on the effectiveness of PES programmes using meta‐analysis where possible. We will use inverse‐variance weighted, random effects model due to anticipated heterogeneity in the included studies (Higgins & Green, 2011). Where there are too few studies, or included studies are considered too heterogeneous in terms of interventions or outcomes, we will report on the individual effect estimates only. We will combine studies using meta‐analysis when we identify two or more effect sizes using a similar outcome construct and where the comparison group state is judged to be similar across the two, similar to the approach taken by Wilson et al. (2011). We will use the metafor package in R software to conduct the meta‐analysis (R Development Core Team, 2008; Viechtbauer, W., 2010).

Once we have identified all included studies we will map out all outcome measures provided in the included studies to determine how we will synthesise outcomes. At a minimum, we plan to synthesise deforestation outcomes and household income, based on the current assessment of the literature. Although we have a loose plan in place to guide this process, we also recognise that the nature of determining which outcome measures will be combined can only be determined *after* we have collected the studies and their corresponding information. Therefore, notably, we will consult with our stakeholders and Advisory Group to help finalize which effects will be synthesised in the same meta‐analytic model.

**Assessment of heterogeneity**

We will assess the heterogeneity of effect sizes graphically using forest plots. We will also assess heterogeneity formally by calculating the Q‐statistic, I^2^, and Tau^2^ to provide an overall estimate of the amount of variability in the distribution of the true effect sizes (Borenstein et al., 2009).

**Moderator analyses**

Depending on the size of the evidence base, we will conduct moderator analysis to explore heterogeneity in the included studies. If feasible, we will use multiple meta‐regression to explore the association between the moderator variables and the outcomes of interest (Borenstein et al., 2009). We will use sub‐group analysis to explore heterogeneity by different treatment sub‐groups. We will undertake the moderator analysis by the following groups of variables:

- Methodology: study design, risk of bias status
- Substantive variables: intervention characteristics (length of programme exposure, size of transfer, type of condition, including whether the PES targets conservation, restoration of an environment or change to a different, more environmentally favourable land use, whether the PES scheme is government, NGO, multilateral / bilateral institution or user financed, and whether it is a national level, regional or local programme), context (region, country income level, tenure security), participant characteristics (gender, socio‐economic status)

**Sensitivity analysis**

We will conduct sensitivity analysis to assess whether the results of the meta‐analysis are sensitive to the removal of any single study. We will do this by removing studies from the meta‐analysis one‐by one and assessing for changes in results.

**Publication bias**

We will attempt to reduce publication bias by searching for and including unpublished studies in the review. In an exploratory manner, we will also test for suggestion of publication bias by using funnel plots and Egger et al.'s (1997) test. Given the inherent subjectivity in assessing funnel plot asymmetry, we will assess sensitivity of meta‐analyses using ‘trim and fill’ (Duvall & Tweedie, 2000), regardless of whether funnel plots suggest asymmetry. Taken together, the totality of these tests will alert us to the possible presence of publication bias.

**Questions 2 and 3: qualitative synthesis**

To address questions 2 and 3 we will complement any statistical meta‐regressions with a qualitative synthesis (Rubenstein et al., 2009). After having completed the detailed coding of all of the included studies as described above, we will re‐review the coding of data on context, intervention design and implementation to identify descriptive findings which remain close to the findings in the primary studies (following Thomas and Harden, 2008). We will then conduct cross‐case analysis (Miles & Huberman, 1994), using a framework based upon the links and assumptions from the program theories of included interventions. We will rank studies by effect size and develop a series of matrices to identify the features of intervention design, implementation and contexts that appear to influence effects.

In case where a sufficient number of studies report detailed qualitative data, we will conduct a thematic synthesis on intervention mechanisms and contexts that mitigate or reinforce intervention effects (Thomas & Harden 2008). In this, we will use inductive coding techniques to identify common descriptive themes based on the reported findings^6^ of the primary studies. We will use EPPI‐Reviewer's coding software to illustrate the link between the inductive codes in the primary studies and the identified descriptive themes. Following the identification of descriptive themes, these will then be configured into higher level analytical themes, which present the results of the thematic synthesis. Analytical themes will be configured around mechanisms and contexts in relation to research question 2 and 3 of this review.

**Questions 4: cost analysis**

Costs and resource use are key considerations in the resource allocation choices of policy‐makers and practitioners. Cost analysis and economic evaluation can help inform decisions about the relative efficiency of environmental programmes (Shemilt et al. 2008; Shemilt et al. 2012). The type cost analyses we will undertake will be determined by the availability and quality of data. Where sufficient data are available, we will assess costs and resource use, and conduct cost‐effectiveness analysis using the formula provided below. We will discuss the limitations in the interpretation and generalisability of the cost‐effectiveness estimates and clearly report all assumptions and underlying calculations used in our cost analysis. If we are unable to calculate cost‐effectiveness ratios we will report costs descriptively based on available data.

If sufficient data is available, we will calculate the cost‐effectiveness ratio using the following formula:

$$CER=\frac{E}{C}$$

Where E is the incremental effect of the intervention on a given outcome, and C is the incremental cost of the intervention.

**Integrated synthesis**

The overarching goal for the review is to provide an integrated synthesis of the findings from synthesis of review questions 1, 2, 3 and 4 in a narrative synthesis. We will use the programme theory provided above to present the findings from the different syntheses with the aim of providing an integrated narrative synthesis addressing the objectives of the review. In doing so we will produce summary of findings tables following the GRADE (Schünemann et al., 2011) and CerQual approaches (Lewin et al., 2015) to facilitate the transparent and systematic presentation of our findings.

## Author(s)

The review will be undertaken jointly by researchers at the International Initiative for Impact Evaluation (3ie) and the Africa Centre for Evidence at the University of Johannesburg. The team also includes a statistician, an information specialist, a substantive expert and an expert in cost‐effectiveness analysis and systematic review methodology.

## Sources of support

The Children's Investment Fund Foundation (CIFF) is providing funding for this review.

## Preliminary timeframe

We plan to submit the draft review report in the first quarter of 2018, with review completion by June 2018 at the latest.

## Footnotes

1 The value of ecosystems services to humans was concretised in the Millennium Ecosystems Assessment report published in 2005 (MEA, 2005). They define ecosystems services as the benefits that humans get from ecosystems.

2 This number is quite high as it is inclusive of a broad range of study designs, including cross‐sectional studies with identification strategies considered to be at a very high risk of bias.

3 An initial list of potential moderating factors are presented in the section, Moderators analyses

4 We will code details of programme design and flag multi‐attribute programmes in the analysis.

5 These criteria draw heavily on Snilstveit et al. 2015

6 We will not use the reported primary data in the included studies, for example interview quotes, as a unit of analysis in the synthesis. We do not expect as sufficient amount of qualitative primary data to be reported in order to allow for a meaningful thematic synthesis.

## Appendix

**CAB Abstracts (Ovid) <1990 to 2017 Week 33>Searched 25th August 2017**

1 (REDD+ or REDD or “Reduced Emissions from Deforestation and Degradation”).ti,ab. (1847)
2 ((pay* or reward* or incentiv* or compensat*) adj10 (agricultur* or livestock or farmland* or farm‐land* or “forest management” or “land management” or technology or conservation or “watershed management” or forest* or deforest* or eco or ecol* or ecos* or environment* or conservation or afforest* or reforest* or restor* or “natural regenerat*” or rainforest* or rain‐forest* or agroforest* or agro‐forest* or “natural resource*” or silvopastor* or “land use*” or “land cover” or “land‐cover” or “land‐use*” or peatland* or peat‐land* or mangrove* or grassland* or grass‐land* or wetland* or wet‐land*)).ti,ab. (15283)
3 (PES or Grain‐for‐green or “Grain for green” or “Sloping Land Conversion Program*” or “Priority Forestry Program*” or “Pago de Servicios Ambientales” or PSA or “Pago por Servicios Ambientales‐Hidrológico” or PSAH).ti,ab. (4576)
4 (sustainability or ecosystem services or carbon sequestration or environmental protection or ecosystem management or biodiversity).sh. and (pay* or reward* or incentiv* or compensat*).ti,ab. (8644)
5 or/1‐4 (24461)
6 (Afghanistan or Albania or Algeria or Angola or Antigua or Barbuda or Argentina or Armenia or Armenian or Aruba or Azerbaijan or Bahrain or Bangladesh or Barbados or Benin or Byelarus or Byelorussian or Belarus or Belorussian or Belorussia or Belize or Bhutan or Bolivia or Bosnia or Herzegovina or Hercegovina or Botswana or Brasil or Brazil or Bulgaria or “Burkina Faso” or “Burkina Fasso” or “Upper Volta” or Burundi or Urundi or Cambodia or “Khmer Republic” or Kampuchea or Cameroon or Cameroons or Cameron or Camerons or “Cape Verde” or “Central African Republic” or Chad or Chile or China or Colombia or Comoros or “Comoro Islands” or Comores or Mayotte or Congo or Zaire or “Costa Rica*” or “Cote d'Ivoire” or “Ivory Coast” or Croatia or Cuba or Czechoslovakia or “Czech Republic” or Slovakia or “Slovak Republic” or Djibouti or “French Somaliland” or Dominica or “Dominican Republic” or “East Timor” or “East Timur” or “Timor Leste” or Ecuador or Egypt or “United Arab Republic” or “El Salvador” or Eritrea or Estonia or Ethiopia or Fiji or Gabon or “Gabonese Republic” or Gambia or Gaza or “Georgia Republic” or “Georgian Republic” or Ghana or “Gold Coast” or Greece or Grenada or Guatemala or Guinea or Guam or Guiana or Guyana or Haiti or Honduras or India or Maldives or Indonesia or Iran or Iraq or Jamaica or Jordan or Kazakhstan or Kazakh or Kenya or Kiribati or Korea or Kosovo or Kyrgyzstan or Kirghizia or “Kyrgyz Republic” or Kirghiz or Kirgizstan or “Lao PDR” or Laos or Latvia or Lebanon or Lesotho or Basutoland or Liberia or Libya or Lithuania or Macedonia or Madagascar or “Malagasy Republic” or Malaysia or Malaya or Malay or Sabah or Sarawak or Malawi or Nyasaland or Mali or Malta or “Marshall Islands” or Mauritania or Mauritius or “Agalega Islands” or Mexico or Micronesia or “Middle East” or Moldova or Moldovia or Moldovian or Mongolia or Montenegro or Morocco or Ifni or Mozambique or Myanmar or Myanma or Burma or Namibia or Nepal or “Netherlands Antilles” or “New Caledonia” or Nicaragua or Niger or Nigeria or “Northern Mariana Islands” or Oman or Muscat or Pakistan or Palau or Palestine or Panama or Paraguay or Peru or Philippines or Philipines or Phillipines or Phillippines or “Puerto Ric*” or Romania or Rumania or Roumania or Russia or Russian or Rwanda or Ruanda or “Saint Kitts” or “St Kitts” or “Nevis” or “Saint Lucia” or “St Lucia” or “Saint Vincent” or “St Vincent” or Grenadines or Samoa or “Samoan Islands” or “Navigator Island” or “Navigator Islands” or “Sao Tome” or “Saudi Arabia” or Senegal or Serbia or Montenegro or Seychelles or “Sierra Leone” or Slovenia or “Sri Lanka” or Ceylon or “Solomon Islands” or Somalia or “South Africa” or Sudan or Suriname or Surinam or Swaziland or Syria or Tajikistan or Tadzhikistan or Tadjikistan or Tadzhik or Tanzania or Thailand or Togo or Togolese Republic or Tonga or Trinidad or Tobago or Tunisia or Turkey or Turkmenistan or Turkmen or Uganda or Ukraine or Uruguay or USSR or “Soviet Union” or “Union of Soviet Socialist Republics” or Uzbekistan or Uzbek or Vanuatu or “New Hebrides” or Venezuela or Vietnam or “Viet Nam” or “West Bank” or Yemen or Yugoslavia or Zambia or Zimbabwe or Rhodesia).mp. not (“African American*” or “African‐American*” or “Mexican American*” or “American Indian*” or “Asian American*” or “native american*”).ti,ab,sh. [mp=abstract, title, original title, broad terms, heading words, identifiers, cabicodes] (1890298)
7 ((developing or “less* developed” or “under developed” or underdeveloped or “under developed” or “middle income” or “low* income”) adj3 (countr* or nation*)).ti,ab. (47918)
8 ((developing or “less* developed” or “under developed” or underdeveloped or “middle income” or “low* income”) adj3 (countr* or nation*)).ti,ab. (47918)
9 ((low adj3 middle adj3 countr*) or Africa or Asia or Caribbean or “West Indies” or “South America” or “Latin America” or “Central America”).ti,ab,sh. (167043)
10 (lmic or lmics or “third world” or “lami countr*” or “transitional countr*”).ti,ab. (2682)
11 or/6‐10 (1960497)
12 (“random* control* trial*” or “random* trial*” or RCT or “propensity score matching” or PSM or “regression discontinuity design” or RDD or “difference in difference*” or matching or (random* adj3 allocat*) or “instrumental variable*” or IV or evaluation or assessment or “comparison group” or counterfactual or “counter factual” or counter‐factual or quasi‐experimental or quasiexperimental or ((quantitative or experiment*) adj3 (design or study or analysis)) or QED).ti,ab,sh. (702156)
13 5 and 11 and 12 (1649)

**Web of Science – Searched 29th August 2017**

# 13 2,222

#12 AND #11 AND #5

Indexes=SCI‐EXPANDED, SSCI, A&HCI, CPCI‐S, CPCI‐SSH, ESCI Timespan=All years

# 12 4,807,089

TS=(“random* control* trial*” or “random* trial*” or RCT or “propensity score matching” or PSM or “regression discontinuity design” or RDD or “difference in difference*” or matching or (random* adj3 allocat*) or “instrumental variable*” or IV or evaluation or assessment or “comparison group” or counterfactual or “counter factual” or counter‐factual or quasi‐experimental or quasiexperimental or ((quantitative or experiment*) NEAR/3 (design or study or analysis)) or QED)

# 11 2,923,401

#10 OR #9 OR #8 OR #7 OR #6

# 10 12,272

TS=(lmic or lmics or “third world” or “lami countr*” or “transitional countr*”)

# 9 487,643

TS=((low NEAR/3 middle NEAR/3 countr*) or Africa or Asia or Caribbean or “West Indies” or “South America” or “Latin America” or “Central America”)

# 8 159,540

TS=((developing or “less* developed” or “under developed” or underdeveloped or “middle income” or “low* income”) NEAR/3 (countr* or nation*))

# 7 159,540

TS=((developing or “less* developed” or “under developed” or underdeveloped or “under developed” or “middle income” or “low* income”) NEAR/3 (countr* or nation*))

# 6 2,627,340

TS=((Afghanistan or Albania or Algeria or Angola or Antigua or Barbuda or Argentina or Armenia or Armenian or Aruba or Azerbaijan or Bahrain or Bangladesh or Barbados or Benin or Byelarus or Byelorussian or Belarus or Belorussian or Belorussia or Belize or Bhutan or Bolivia or Bosnia or Herzegovina or Hercegovina or Botswana or Brasil or Brazil or Bulgaria or “Burkina Faso” or “Burkina Fasso” or “Upper Volta” or Burundi or Urundi or Cambodia or “Khmer Republic” or Kampuchea or Cameroon or Cameroons or Cameron or Camerons or “Cape Verde” or “Central African Republic” or Chad or Chile or China or Colombia or Comoros or “Comoro Islands” or Comores or Mayotte or Congo or Zaire or “Costa Rica*” or “Cote d'Ivoire” or “Ivory Coast” or Croatia or Cuba or Czechoslovakia or “Czech Republic” or Slovakia or “Slovak Republic” or Djibouti or “French Somaliland” or Dominica or “Dominican Republic” or “East Timor” or “East Timur” or “Timor Leste” or Ecuador or Egypt or “United Arab Republic” or “El Salvador” or Eritrea or Estonia or Ethiopia or Fiji or Gabon or “Gabonese Republic” or Gambia or Gaza or “Georgia Republic” or “Georgian Republic” or Ghana or “Gold Coast” or Greece or Grenada or Guatemala or Guinea or Guam or Guiana or Guyana or Haiti or Honduras or India or Maldives or Indonesia or Iran or Iraq or Jamaica or Jordan or Kazakhstan or Kazakh or Kenya or Kiribati or Korea or Kosovo or Kyrgyzstan or Kirghizia or “Kyrgyz Republic” or Kirghiz or Kirgizstan or “Lao PDR” or Laos or Latvia or Lebanon or Lesotho or Basutoland or Liberia or Libya or Lithuania or Macedonia or Madagascar or “Malagasy Republic” or Malaysia or Malaya or Malay or Sabah or Sarawak or Malawi or Nyasaland or Mali or Malta or “Marshall Islands” or Mauritania or Mauritius or “Agalega Islands” or Mexico or Micronesia or “Middle East” or Moldova or Moldovia or Moldovian or Mongolia or Montenegro or Morocco or Ifni or Mozambique or Myanmar or Myanma or Burma or Namibia or Nepal or “Netherlands Antilles” or “New Caledonia” or Nicaragua or Niger or Nigeria or “Northern Mariana Islands” or Oman or Muscat or Pakistan or Palau or Palestine or Panama or Paraguay or Peru or Philippines or Philipines or Phillipines or Phillippines or “Puerto Ric*” or Romania or Rumania or Roumania or Russia or Russian or Rwanda or Ruanda or “Saint Kitts” or “St Kitts” or “Nevis” or “Saint Lucia” or “St Lucia” or “Saint Vincent” or “St Vincent” or Grenadines or Samoa or “Samoan Islands” or “Navigator Island” or “Navigator Islands” or “Sao Tome” or “Saudi Arabia” or Senegal or Serbia or Montenegro or Seychelles or “Sierra Leone” or Slovenia or “Sri Lanka” or Ceylon or “Solomon Islands” or Somalia or “South Africa” or Sudan or Suriname or Surinam or Swaziland or Syria or Tajikistan or Tadzhikistan or Tadjikistan or Tadzhik or Tanzania or Thailand or Togo or Togolese Republic or Tonga or Trinidad or Tobago or Tunisia or Turkey or Turkmenistan or Turkmen or Uganda or Ukraine or Uruguay or USSR or “Soviet Union” or “Union of Soviet Socialist Republics” or Uzbekistan or Uzbek or Vanuatu or “New Hebrides” or Venezuela or Vietnam or “Viet Nam” or “West Bank” or Yemen or Yugoslavia or Zambia or Zimbabwe or Rhodesia) NOT (“AfricanAmerican*” or “African‐American*” or “Mexican American*” or “American Indian*” or “Asian American*” or “native american*”))

# 5 89,570

#4 OR #3 OR #2 OR #1

# 4 11,393

TS=((sustainability or “ecosystem services” or “carbon sequestration” or “environmental protection” or “ecosystem management” or biodiversity) AND (pay* or reward* or incentiv* or compensat*))

# 3 52,400

TS=(PES or Grain‐for‐green or “Grain for green” or “Sloping Land Conversion Program*” or “Priority Forestry Program*” or “Pago de Servicios Ambientales” or PSA or “Pago por Servicios Ambientales‐Hidrológico” or PSAH)

# 2 29,051

TS=((pay* or reward* or incentiv* or compensat*) NEAR/10 (agricultur* or livestock or farmland* or farm‐land* or “forest management” or “land management” or technology or conservation or “watershed management” or forest* or deforest* or eco or ecol* or ecos* or environment* or conservation or afforest* or reforest* or restor* or “natural regenerat*” or rainforest* or rain‐forest* or agroforest* or agro‐forest* or “natural resource*” or silvopastor* or “land use*” or “land cover” or “land‐cover” or “land‐use*” or peatland* or peat‐land* or mangrove* or grassland* or grass‐land* or wetland* or wet‐land*))

# 1 2,350

TS=(REDD+ or REDD or “Reduced Emissions from Deforestation and Degradation”)

**Ebsco Discovery – Agris, Econlit & RePeC – Searched 30th August 2017**

**Greenfile (Ebsco) ‐ Searched 30th August 2017**

S12 S5 AND S10 AND S11 9,445 (**Agris – 815; Econlit – 230; RePeC – 412; Greenfile ‐ 295**)

S11 TI ((“random* control* trial*” or “random* trial*” or RCT or “propensity score matching” or PSM or “regression discontinuity design” or RDD or “difference in difference*” or matching or (random* N3 allocat*) or “instrumental variable*” or IV or evaluation or assessment or “comparison group” or counterfactual or “counter factual” or counter‐factual or quasi‐experimental or quasiexperimental or ((quantitative or experiment*) N3 (design or study or analysis)) or QED)) OR AB ((“random* control* trial*” or “random* trial*” or RCT or “propensity score matching” or PSM or “regression discontinuity design” or RDD or “difference in difference*” or matching or (random* N3 allocat*) or “instrumental variable*” or IV or evaluation or assessment or “comparison group” or counterfactual or “counter factual” or counter‐factual or quasi‐experimental or quasiexperimental or ((quantitative or experiment*) N3 (design or study or analysis)) or QED)) OR SU ((“random* control* trial*” or “random* trial*” or RCT or “propensity score matching” or PSM or “regression discontinuity design” or RDD or “difference in difference*” or matching or (random* N3 allocat*) or “instrumental variable*” or IV or evaluation or assessment or “comparison group” or counterfactual or “counter factual” or counter‐factual or quasi‐experimental or quasiexperimental or ((quantitative or experiment*) N3 (design or study or analysis)) or QED))

18,629,561

S10 S6 OR S7 OR S8 OR S9 25,507,282

S9 TI ((lmic or lmics or “third world” or “lami countr*” or “transitional countr*”)) OR AB ((lmic or lmics or “third world” or “lami countr*” or “transitional countr*”)) OR SU ((lmic or lmics or “third world” or “lami countr*” or “transitional countr*”)) 117,221

S8 TI (((low N3 middle N3 countr*) or Africa or Asia or Caribbean or “West Indies” or “South America” or “Latin America” or “Central America”)) OR AB (((low N3 middle N3 countr*) or Africa or Asia or Caribbean or “West Indies” or “South America” or “Latin America” or “Central America”)) OR SU (((low N3 middle N3 countr*) or Africa or Asia or Caribbean or “West Indies” or “South America” or “Latin America” or “Central America”)) 6,962,117

S7 TI (((developing or “less* developed” or “under developed” or underdeveloped or “middle income” or “low* income”) N3 (countr* or nation*))) OR AB (((developing or “less* developed” or “under developed” or underdeveloped or “middle income” or “low* income”) N3 (countr* or nation*))) OR SU (((developing or “less* developed” or “under developed” or underdeveloped or “middle income” or “low* income”) N3 (countr* or nation*))) 1,738,088

S6 TI ((Afghanistan or Albania or Algeria or Angola or Antigua or Barbuda or Argentina or Armenia or Armenian or Aruba or Azerbaijan or Bahrain or Bangladesh or Barbados or Benin or Byelarus or Byelorussian or Belarus or Belorussian or Belorussia or Belize or Bhutan or Bolivia or Bosnia or Herzegovina or Hercegovina or Botswana or Brasil or Brazil or Bulgaria or “Burkina Faso” or “Burkina Fasso” or “Upper Volta” or Burundi or Urundi or Cambodia or “Khmer Republic” or Kampuchea or Cameroon or Cameroons or Cameron or Camerons or “Cape Verde” or “Central African Republic” or Chad or Chile or China or Colombia or Comoros or “Comoro Islands” or Comores or Mayotte or Congo or Zaire or “Costa Rica*” or “Cote d'Ivoire” or “Ivory Coast” or Croatia or Cuba or Czechoslovakia or “Czech Republic” or Slovakia or “Slovak Republic” or Djibouti or “French Somaliland” or Dominica or “Dominican Republic” or “East Timor” or “East Timur” or “Timor Leste” or Ecuador or Egypt or “United Arab Republic” or “El Salvador” or Eritrea or Estonia or Ethiopia or Fiji or Gabon or “Gabonese Republic” or Gambia or Gaza or “Georgia Republic” or “Georgian Republic” or Ghana or “Gold Coast” or Greece or Grenada or Guatemala or Guinea or Guam or Guiana or Guyana or Haiti or Honduras or India or Maldives or Indonesia or Iran or Iraq or Jamaica or Jordan or Kazakhstan or Kazakh or Kenya or Kiribati or Korea or Kosovo or Kyrgyzstan or Kirghizia or “Kyrgyz Republic” or Kirghiz or Kirgizstan or “Lao PDR” or Laos or Latvia or Lebanon or Lesotho or Basutoland or Liberia or Libya or Lithuania or Macedonia or Madagascar or “Malagasy Republic” or Malaysia or Malaya or Malay or Sabah or Sarawak or Malawi or Nyasaland or Mali or Malta or “Marshall Islands” or Mauritania or Mauritius or “Agalega Islands” or Mexico or Micronesia or “Middle East” or Moldova or Moldovia or Moldovian or Mongolia or Montenegro or Morocco or Ifni or Mozambique or Myanmar or Myanma or Burma or Namibia or Nepal or “Netherlands Antilles” or “New Caledonia” or Nicaragua or Niger or Nigeria or “Northern Mariana Islands” or Oman or Muscat or Pakistan or Palau or Palestine or Panama or Paraguay or Peru or Philippines or Philipines or Phillipines or Phillippines or “Puerto Ric*” or Romania or Rumania or Roumania or Russia or Russian or Rwanda or Ruanda or “Saint Kitts” or “St Kitts” or “Nevis” or “Saint Lucia” or “St Lucia” or “Saint Vincent” or “St Vincent” or Grenadines or Samoa or “Samoan Islands” or “Navigator Island” or “Navigator Islands” or “Sao Tome” or “Saudi Arabia” or Senegal or Serbia or Montenegro or Seychelles or “Sierra Leone” or Slovenia or “Sri Lanka” or Ceylon or “Solomon Islands” or Somalia or “South Africa” or Sudan or Suriname or Surinam or Swaziland or Syria or Tajikistan or Tadzhikistan or Tadjikistan or Tadzhik or Tanzania or Thailand or Togo or “Togolese Republic” or Tonga or Trinidad or Tobago or Tunisia or Turkey or Turkmenistan or Turkmen or Uganda or Ukraine or Uruguay or USSR or “Soviet Union” or “Union of Soviet Socialist Republics” or Uzbekistan or Uzbek or Vanuatu or “New Hebrides” or Venezuela or Vietnam or “Viet Nam” or “West Bank” or Yemen or Yugoslavia or Zambia or Zimbabwe or Rhodesia) NOT (“African American*” or “African‐American*” or “Mexican American*” or “American Indian*” or “Asian American*” or “native american*”)) OR AB ((Afghanistan or Albania or Algeria or Angola or Antigua or Barbuda or Argentina or Armenia or Armenian or Aruba or Azerbaijan or Bahrain or Bangladesh or Barbados or Benin or Byelarus or Byelorussian or Belarus or Belorussian or Belorussia or Belize or Bhutan or Bolivia or Bosnia or Herzegovina or Hercegovina or Botswana or Brasil or Brazil or Bulgaria or “Burkina Faso” or “Burkina Fasso” or “Upper Volta” or Burundi or Urundi or Cambodia or “Khmer Republic” or Kampuchea or Cameroon or Cameroons or Cameron or Camerons or “Cape Verde” or “Central African Republic” or Chad or Chile or China or Colombia or Comoros or “Comoro Islands” or Comores or Mayotte or Congo or Zaire or “Costa Rica*” or “Cote d'Ivoire” or “Ivory Coast” or Croatia or Cuba or Czechoslovakia or “Czech Republic” or Slovakia or “Slovak Republic” or Djibouti or “French Somaliland” or Dominica or “Dominican Republic” or “East Timor” or “East Timur” or “Timor Leste” or Ecuador or Egypt or “United Arab Republic” or “El Salvador” or Eritrea or Estonia or Ethiopia or Fiji or Gabon or “Gabonese Republic” or Gambia or Gaza or “Georgia Republic” or “Georgian Republic” or Ghana or “Gold Coast” or Greece or Grenada or Guatemala or Guinea or Guam or Guiana or Guyana or Haiti or Honduras or India or Maldives or Indonesia or Iran or Iraq or Jamaica or Jordan or Kazakhstan or Kazakh or Kenya or Kiribati or Korea or Kosovo or Kyrgyzstan or Kirghizia or “Kyrgyz Republic” or Kirghiz or Kirgizstan or “Lao PDR” or Laos or Latvia or Lebanon or Lesotho or Basutoland or Liberia or Libya or Lithuania or Macedonia or Madagascar or “Malagasy Republic” or Malaysia or Malaya or Malay or Sabah or Sarawak or Malawi or Nyasaland or Mali or Malta or “Marshall Islands” or Mauritania or Mauritius or “Agalega Islands” or Mexico or Micronesia or “Middle East” or Moldova or Moldovia or Moldovian or Mongolia or Montenegro or Morocco or Ifni or Mozambique or Myanmar or Myanma or Burma or Namibia or Nepal or “Netherlands Antilles” or “New Caledonia” or Nicaragua or Niger or Nigeria or “Northern Mariana Islands” or Oman or Muscat or Pakistan or Palau or Palestine or Panama or Paraguay or Peru or Philippines or Philipines or Phillipines or Phillippines or “Puerto Ric*” or Romania or Rumania or Roumania or Russia or Russian or Rwanda or Ruanda or “Saint Kitts” or “St Kitts” or “Nevis” or “Saint Lucia” or “St Lucia” or “Saint Vincent” or “St Vincent” or Grenadines or Samoa or “Samoan Islands” or “Navigator Island” or “Navigator Islands” or “Sao Tome” or “Saudi Arabia” or Senegal or Serbia or Montenegro or Seychelles or “Sierra Leone” or Slovenia or “Sri Lanka” or Ceylon or “Solomon Islands” or Somalia or “South Africa” or Sudan or Suriname or Surinam or Swaziland or Syria or Tajikistan or Tadzhikistan or Tadjikistan or Tadzhik or Tanzania or Thailand or Togo or “Togolese Republic” or Tonga or Trinidad or Tobago or Tunisia or Turkey or Turkmenistan or Turkmen or Uganda or Ukraine or Uruguay or USSR or “Soviet Union” or “Union of Soviet Socialist Republics” or Uzbekistan or Uzbek or Vanuatu or “New Hebrides” or Venezuela or Vietnam or “Viet Nam” or “West Bank” or Yemen or Yugoslavia or Zambia or Zimbabwe or Rhodesia) NOT (“African American*” or “African‐American*” or “Mexican American*” or “American Indian*” or “Asian American*” or “native american*”)) OR SU ((Afghanistan or Albania or Algeria or Angola or Antigua or Barbuda or Argentina or Armenia or Armenian or Aruba or Azerbaijan or Bahrain or Bangladesh or Barbados or Benin or Byelarus or Byelorussian or Belarus or Belorussian or Belorussia or Belize or Bhutan or Bolivia or Bosnia or Herzegovina or Hercegovina or Botswana or Brasil or Brazil or Bulgaria or “Burkina Faso” or “Burkina Fasso” or “Upper Volta” or Burundi or Urundi or Cambodia or “Khmer Republic” or Kampuchea or Cameroon or Cameroons or Cameron or Camerons or “Cape Verde” or “Central African Republic” or Chad or Chile or China or Colombia or Comoros or “Comoro Islands” or Comores or Mayotte or Congo or Zaire or “Costa Rica*” or “Cote d'Ivoire” or “Ivory Coast” or Croatia or Cuba or Czechoslovakia or “Czech Republic” or Slovakia or “Slovak Republic” or Djibouti or “French Somaliland” or Dominica or “Dominican Republic” or “East Timor” or “East Timur” or “Timor Leste” or Ecuador or Egypt or “United Arab Republic” or “El Salvador” or Eritrea or Estonia or Ethiopia or Fiji or Gabon or “Gabonese Republic” or Gambia or Gaza or “Georgia Republic” or “Georgian Republic” or Ghana or “Gold Coast” or Greece or Grenada or Guatemala or Guinea or Guam or Guiana or Guyana or Haiti or Honduras or India or Maldives or Indonesia or Iran or Iraq or Jamaica or Jordan or Kazakhstan or Kazakh or Kenya or Kiribati or Korea or Kosovo or Kyrgyzstan or Kirghizia or “Kyrgyz Republic” or Kirghiz or Kirgizstan or “Lao PDR” or Laos or Latvia or Lebanon or Lesotho or Basutoland or Liberia or Libya or Lithuania or Macedonia or Madagascar or “Malagasy Republic” or Malaysia or Malaya or Malay or Sabah or Sarawak or Malawi or Nyasaland or Mali or Malta or “Marshall Islands” or Mauritania or Mauritius or “Agalega Islands” or Mexico or Micronesia or “Middle East” or Moldova or Moldovia or Moldovian or Mongolia or Montenegro or Morocco or Ifni or Mozambique or Myanmar or Myanma or Burma or Namibia or Nepal or “Netherlands Antilles” or “New Caledonia” or Nicaragua or Niger or Nigeria or “Northern Mariana Islands” or Oman or Muscat or Pakistan or Palau or Palestine or Panama or Paraguay or Peru or Philippines or Philipines or Phillipines or Phillippines or “Puerto Ric*” or Romania or Rumania or Roumania or Russia or Russian or Rwanda or Ruanda or “Saint Kitts” or “St Kitts” or “Nevis” or “Saint Lucia” or “St Lucia” or “Saint Vincent” or “St Vincent” or Grenadines or Samoa or “Samoan Islands” or “Navigator Island” or “Navigator Islands” or “Sao Tome” or “Saudi Arabia” or Senegal or Serbia or Montenegro or Seychelles or “Sierra Leone” or Slovenia or “Sri Lanka” or Ceylon or “Solomon Islands” or Somalia or “South Africa” or Sudan or Suriname or Surinam or Swaziland or Syria or Tajikistan or Tadzhikistan or Tadjikistan or Tadzhik or Tanzania or Thailand or Togo or “Togolese Republic” or Tonga or Trinidad or Tobago or Tunisia or Turkey or Turkmenistan or Turkmen or Uganda or Ukraine or Uruguay or USSR or “Soviet Union” or “Union of Soviet Socialist Republics” or Uzbekistan or Uzbek or Vanuatu or “New Hebrides” or Venezuela or Vietnam or “Viet Nam” or “West Bank” or Yemen or Yugoslavia or Zambia or Zimbabwe or Rhodesia) NOT (“African American*” or “African‐American*” or “Mexican American*” or “American Indian*” or “Asian American*” or “native american*”)) 23,130,617

S5 S1 OR S2 OR S3 OR S4 435,257

S4 TI ((sustainability or “ecosystem services” or “carbon sequestration” or “environmental protection” or “ecosystem management” or biodiversity) N5 (pay* or reward* or incentiv* or compensat*)) OR AB ((sustainability or “ecosystem services” or “carbon sequestration” or “environmental protection” or “ecosystem management” or biodiversity) N5 (pay* or reward* or incentiv* or compensat*)) OR SU ((sustainability or “ecosystem services” or “carbon sequestration” or “environmental protection” or “ecosystem management” or biodiversity) N5 (pay* or reward* or incentiv* or compensat*)) 12,309

S3 TI (PES or Grain‐for‐green or “Grain for green” or “Sloping Land Conversion Program*” or “Priority Forestry Program*” or “Pago de Servicios Ambientales” or PSA or “Pago por Servicios Ambientales‐Hidrológico” or PSAH) OR AB (PES or Grain‐for‐green or “Grain for green” or “Sloping Land Conversion Program*” or “Priority Forestry Program*” or “Pago de Servicios Ambientales” or PSA or “Pago por Servicios Ambientales‐Hidrológico” or PSAH) OR SU (PES or Grain‐for‐green or “Grain for green” or “Sloping Land Conversion Program*” or “Priority Forestry Program*” or “Pago de Servicios Ambientales” or PSA or “Pago por Servicios Ambientales‐Hidrológico” or PSAH) 157,030

S2 TI (((pay* or reward* or incentiv* or compensat*) N10 (agricultur* or livestock or farmland* or farm‐land* or “forest management” or “land management” or technology or conservation or “watershed management” or forest* or deforest* or eco or ecol* or ecos* or environment* or conservation or afforest* or reforest* or restor* or “natural regenerat*” or rainforest* or rain‐forest* or agroforest* or agro‐forest* or “natural resource*” or silvopastor* or “land use*” or “land cover” or “land‐cover” or “land‐use*” or peatland* or peat‐land* or mangrove* or grassland* or grass‐land* or wetland* or wet‐land*))) OR AB (((pay* or reward* or incentiv* or compensat*) N10 (agricultur* or livestock or farmland* or farm‐land* or “forest management” or “land management” or technology or conservation or “watershed management” or forest* or deforest* or eco or ecol* or ecos* or environment* or conservation or afforest* or reforest* or restor* or “natural regenerat*” or rainforest* or rain‐forest* or agroforest* or agro‐forest* or “natural resource*” or silvopastor* or “land use*” or “land cover” or “land‐cover” or “land‐use*” or peatland* or peat‐land* or mangrove* or grassland* or grass‐land* or wetland* or wet‐land*))) OR SU (((pay* or reward* or incentiv* or compensat*) N10 (agricultur* or livestock or farmland* or farm‐land* or “forest management” or “land management” or technology or conservation or “watershed management” or forest* or deforest* or eco or ecol* or ecos* or environment* or conservation or afforest* or reforest* or restor* or “natural regenerat*” or rainforest* or rain‐forest* or agroforest* or agro‐forest* or “natural resource*” or silvopastor* or “land use*” or “land cover” or “land‐cover” or “land‐use*” or peatland* or peat‐land* or mangrove* or grassland* or grass‐land* or wetland* or wet‐land*))) 243,445

S1 TI (REDD+ or REDD or “Reduced Emissions from Deforestation and Degradation”) OR AB (REDD+ or REDD or “Reduced Emissions from Deforestation and Degradation”) OR SU (REDD+ or REDD or “Reduced Emissions from Deforestation and Degradation”) 16,106

**AgEcon – Searched 30th August 2017**

((pay* OR reward* OR incentiv* OR compensat*) AND (agricultur* OR livestock OR farmland* OR farm‐land* OR “forest management” OR “land management” OR technology OR conservation OR “watershed management” OR forest* OR deforest* OR eco OR ecol* OR ecos* OR environment* OR conservation OR afforest* OR reforest* OR restor* OR “natural regenerat*” OR rainforest* OR rain‐forest* OR agroforest* OR agro‐forest* OR “natural resource*” OR silvopastor* OR “land use*” OR “land cover” OR “land‐cover” OR “land‐use*” OR peatland* OR peat‐land* OR mangrove* OR grassland* OR grass‐land* OR wetland* OR wet‐land*))

## Appendix

**Table:** Intervention and study description, process, implementation, qualitative and cost data

	**Description**	**Question**	**Coding**
**Report identification**	Unique study identification #		E.g. PES001
	First author ‐ impact evaluation	Surname	Surname
	Other papers used for coding	First author surname and type of paper of any qualitative, descriptive quantitative, process evaluations or project documents used for coding	
	General comments	**(1) General comments** Any general comments on study not coded elsewhere **(2) Issues of comparability** Please report any potential issues of comparability between different documents (e.g. different documents assess a programme/intervention at different scales [geographic/time scale]). If the issue of comparability related only to a certain secion of a document (e.g. cost data), please put in brackets in relevant cell.	Open answer
	Publication date	Year (letter)	XXXX (a)
	Publication type	What is the impact evaluation publication type?	1= Peer‐reviewed journal 2= Book chapter/book 3= Conference paper 4= Organisation report 5= Working paper 6= Implementation document 7= other grey 8= PhD thesis / dissertation
	Funding agency	Who is funding the evaluation/study?	1= Public institution (e.g. govt, NGO, university, research institute) 2= Private institution (e.g. private company) 3= Multilateral Organisation (World Bank, UN) 4 = Foundations 8= Not clear 9= Not applicable (Non‐funded)
	Name of funding agency	Please add name of the agency funding the evaluation	Open answer
	Independence of evaluation	What level of independence is there between the impleenting agency and study team?	1=Funding and author team independent of implementers/ funders of programme 2=Funding independent of implementers/ funders of programme, but includes authors from funder/ implementer 3=Evaluation funded and undetaken by funders/ implementers 8=Unclear
	Independent data collection	Has the data been collected by an independent party?	1= Yes 2=No 8=Not clear
	Conflict of interest	Is there a potential conflict of interest associated with study which could influence results collected/reported? (eg. Is there a declaration of conflict of interest? Is any of the authors related in any way to the funding or implenting institution?)	1=Yes 2=No 8=Not clear
	Comments on conflict of interest	Please add reason for your answer to whether there is a conflict of interest.	Open answer
	Language of publication	Language of publication of the impact evaluation, e.g. Spanish, English etc.	Open answer
	Other methods	If the impact evaluation addresses other questions than effectiveness note questions and methods used here.	Open answer (this will include for example mixed‐methods to assess implementation, adherence, participant views etc)
**Intervention descriptives**	Programme or project name	State the programme or project name. If no name, then list the location (e.g. Town, village etc.).	Open answer
	Intervention type	Indicate type of intervention	1 = PES alone 2 = PES + other intervention
	Type of ecosystem targeted	Indicate the type of ecosystem targeted	1 = Forests 2 = Farmland 3 = Grassland 4 = Mangroves 5 = Wetlands
	Intervention description	Provide descriptive details about the intervention. *Include detail on any other intervention provided alongside the PES, including alternative livelihoods strategies, awareness raising activities, increased forest monitoring etc.*	Open answer
	Objectives of intervention	Type of objective(s) of intervention	1=Conservation only 2=Restoration 3=Environmentally beneficial/ preferable to BAU land‐use 4= Socioeconomic (livelihoods, poverty reduction etc) 5=Other (add description in comments)
	Objectives of intervention	State any objectives stated in study or project document, including whether the study targets both environmental and poverty objectives.	
	Size of payment	Indicate the size of the regular payment	Open answer, $
	Frequency of payment	Indicate how frequently the payment is made (annual, monthly, etc).	Open answer
	Method of payment	Indicate how payment made to participants	Open answer
	Conditionality	Indicate the stated conditions of the PES programme	Open answer
	Intervention scale	What is the scale of the intervention?	1=Local 2=Regional 2=National
	Intervention implementing agency	Who is implementing the intervention? State the name (and department) of the implementing agency.	Open answer
	Intervention funding agency	Type of funder	1=Government 2=User financed (companies using env service) 3=NGO 4=Multilateral/bilater organisation 5=Carbon offset mechanism 6=Other
	Intervention funding agency	Name of intervention funding agency	Open answer
	Intervention target group	What were the characteristics of beneficiaries used to target the intervention?	Open answer
	Targeting methods	How were beneficiaries targeted for the programme (Eg: how was the targeting implemented)?	Open answer
	Intervention start	Start date (if not stated, state study date) of intervention	XX/XXXX
	Intervention end	State end date (if ongoing state ongoing)	XX/XXXX
	Follow up	How long after the last payment was outcome data collected?	indicate number of months (numerical only). If not clear state so
	Program theory	Do the authors make explicit reference to program theory, theory of change or similar?	1=Yes 2=No 8=Not clear
	Program theory	Report any description/statement of program theory as stated by author(s).	Open answer
**Context**	Country	List countries the study was conducted in	Country 1, Country 2, etc.
	Detailed location	If provided, give detailed information on where the study took place within a country, for example regions/districts covered	Open answer
	World Bank Region	Select region(s) the study was conducted in according to World Bank. For more info on region classification see http://data.worldbank.org/country	1= East Asia & Pacific 2= Europe & Central Asia 3= Latin America & Caribbean 4= Middle East & North Africa 5=South Asia 6=Sub‐Saharan Africa
	WB Income category	Select the World Bank income classification of the country at the time of the study	1 = Low income country 2 = Lower‐middle income country 3 = Upper‐middle income country
	REDD+ status	Is the country where the evaluation took place a REDD+ country?	1= Yes, 2 = No, 3 = Unclear
	Environmental performance index	How does the country rank on the Environmental Perfomance Index: http://epi.yale.edu/?	Open answer ‐ to be filled in after coding complete
	Baseline deforestation rates	Report any data / description on deforestation rates in programme / comparison area	Open answer
	Baseline socio‐economic status of participants	Report any data / description on baseline socio‐economic status of participants	Open answer
	Property right regime	Report any description in the primary evaluation or qualitative documents of the existing property rights regime	Open answer
**Process and implementation**	Information about program take‐up/adherence (among beneficiaries)	Is there any information about program take‐up/adherence (among beneficiaries)? Commentary by authors should be used when information on program adherence etc. is not backed up by some sort of research / when the authors do not report that/how they collected data to assess these areas.	1=Yes, commentary from author; 2=No; 4= Yes, formally assessed
	Methods of assessing take‐up/adherence	Which methods are used to assess program take‐up/adherence?	1= Observation by intervention staff 2= Reporting by participants 3= Other 4= Commentary from author 9= Not measured
	Results of the assessment of take‐up/adherence	What is the result/ information provided of the assessment of program take‐up/adherence?	Open answer
	Information about implementation fidelity / service delivery quality	Is there any information on implementation fidelity/ service delivery quality? Commentary by authors should be used when information on program adherence etc. is not backed up by some sort of research / when the authors do not report that/how they collected data to assess these areas.	1=Yes, commentary from author; 2=No; 4= Yes, formally assessed
	Methods of assessing intervention fidelity	Which methods are used to assess implementation fidelity/ service delivery quality	1= Observation by intervention staff 2= Reporting by participants 3= Other 4= Commentary from author 9= Not measured
	Results of the assessment of intervention fidelity	What is the result/ information provided of the assessment of implementation fidelity/ service delivery quality	Open answer
	Other description of process factors	Any other description of process factors not covered above	Open answer
	Barriers and facilitators	Do the study identify any barriers and facilitators not included above?	Open answer
**Cost**	Cost	Are any unit cost data / cost‐effectiveness estimates provided?	1=Yes 2=No
	Cost details	If yes, report any details of unit cost and/or total cost. Please also report year and currency.	Open answer
**External Validity**	Length of study	Length of study in months (Where study length not reported, code as length of intervention, noting that in brackets)	# months, if not reported N/A
	Efficacy or effectiveness trial	Was the intervention implemented under “real world” conditions? By real world we mean a programme implemented independently of the evaluation, either by government, NGO or international agency. Eg: the programme is not designed and implemented for the purpose of research	1=Yes 2=No 9= N/A
	Personell implementing the programme	Who was in charge of implementing the program?	1=PI/ researchers (study authors); 2= implementing agency staff, 3= external agency (eg: survey firm); 4=Others; 8= Not clear
	Sampling frame for the study	State the sampling frame (list of all those within a population who can be sampled, ie. households, communities) for selection of study participants (i.e. Census, etc).	Open answer
	Author discussion of external validity	Do the authors discuss or explicitly address generalisability / applicability?	Open answer
	Theory	Is there any reference to theory of change underlying intervention?	1=Yes 2=No 9= N/A
	Theory based evaluation	Is the study using theory to inform the evaluation design and analysis?	Open answer ‐ describe if and how the authors use theory in the evaluation. Do they for example use it to inform data collection? Do they do any causal chain analysis?
**Equity**	Consideration of equity	Does the study consider equity?	1=Yes 2=No
	Equity methods	How does the study consider equity?	1=intervention target a disadvantaged group 2=study measures inequality 3=sub‐group analysis by dimension of inequity
	Equity dimension	What dimension(s) of equity does the study consider?	1=gender 2=socioeconomic status 3=place of recidence 4=land ownership 5=landsize

**Table:** Effect size data

	Description	Question	Coding
ID	Unique study identification #		E.g. PES001
	First author ‐ impact evaluation	Surname	Open answer
Outcome for effect size (answer for all studies)	Primary outcome	Which primary outcomes is being coded?	1 = Forest cover / deforestation 2 = Forest condition 3 = Carbon stocks 4 = Greenhouse gas emissions 5 = Income / consumption / expenditure 6 = Food security 7 = Other socio‐economic outcome 8 = Intermediate outcomes
	Sub‐group analysis	Is this effect size data for a sub‐group?	1 = No 2 = Yes
	Sub‐group analysis decription	If yes to question 2, which type of sub‐group?	Open answer ‐ this can include separate samples for gender, income, place of residence, land size, head of household (eg: female or male headed)
	Definition of outcome	Please provide the authors definition of the outcome (including description of the sub‐group if relevant)	Open answer
	Effect size location	Which page(s) contain the effect size data?	Open answer
	Data to be extracted	Which type of data to be extracted?	1 = Continuous ‐ means and SDs 2 = Continuous ‐ mean difference and SD 2 = Dichotomous outcome ‐ proportions 3 = Regression data
Effect size data (answer for all studies)	Sample size metric	Sample size unit of analysis	1= Individual 2= Household 3= Group (e.g. community organisation) 4= Plot 5= Village 6=Not clear
	Treatment effect estimated	What treatment effect is estimated?	1=ITT 2=ATET 3=ATE 4=LATE
	Sample size (treatment)	Initial sample size treatment group	#
	Sample size (control)	Initial sample size control group	#
	Sample size (total)	Initial sample size total	#
	Observations (treatment)	Number of treatment observations after attrition (individuals)	#
	Observations (control)	Number of control observations after attrition (individuals)	#
	Observations (total)	Total number of control observations after attrition (individuals)	#
Outcome data ‐ if continuous (Means and SDs)	Baseline outcome treatement	State result of baseline outcome for treatment group	#
	SD Baseline outcome treatement	State SD of baseline outcome measure for treatment group	#
	Sample size baseline treatment	State sample size at baseline	#
	Baseline outcome control	State result of baseline outcome for control group	#
	SD Baseline outcome control	State SD of baseline outcome measure for contol group	#
	Sample size baseline control	State sample size at baseline	#
	Outcome in treatment post intervention	State result of post intervention outcome for treatment group	#
	SD Outcome in treatment post intervention	State SD of post intervention outcome measure for treatment group	#
	Number with outcome in treatment post intervention	State sample size post intervention	#
	Outcome in control post intervention	State result of post intervention outcome for control group	#
	SD Outcome in control post intervention	State SD of post intervention outcome measure for control group	#
	Number with outcome in contol post intervention	State sample size post intervention	#
	Outcome in treatment 1st follow up	State result of 1st follow up outcome measure for treatment group	#
	SD Outcome in treatment 1st follow up	State SD 1st follow up outcome measure for treatment group	#
	Number with outcome in treatment 1st follow up	State sample size first follow up	#
	Outcome in control 1st follow up	State result of 1st follow up outcome measure for treatment group	#
	SD Outcome in control 1st follow up	State SD 1st follow up outcome measure for treatment group	#
	Number with outcome in control 1st follow up	State sample size first follow up	#
Outcome data ‐ If continuous (Mean difference and SD at follow up)	Mean difference at follow up	State mean difference	#
	SD at follow up	State SD at follow up	#
Outcomes data ‐ if dichotomous (Proportions r)	Baseline number with outcome in treatement	State result of baseline outcome for treatment group	#
	Sample size baseline treatment	State sample size at baseline	#
	Proportion with outcome at baseline in treatment	State proportion with outcome at baseline in treatment	#
	Baseline number with outcome in control	State result of baseline outcome for treatment group	#
	Sample size baseline control	State sample size at baseline	#
	Proportion with outcome at baseline in control	State proportion with outcome at baseline in contol	#
	Number with outcome in treatment post intervention	State number with outcome post intervention for treatment group	#
	Sample size post intervention treatment	State sample size for treatment group post intervention	#
	Proportion with outcome in treatment group post intervention	State proportion with outcome post intervention in control group	#
	Number with outcome in control post intervention	State number with outcome post intervention for control group	#
	Sample size post intervention control	State sample size for control group post intervention	#
	Proportion with outcome in control group post intervention	State proportion with outcome post intervention in control group	#
	Number with outcome in treatment 1st follow up	State number with outcome at 1st follow up for treatment group	#
	Sample size 1st follow up treatment	State sample size at 1st follow up for treatment group	#
	Proportion with outcome in treatment group 1st follow up	State proportion with outcome at 1st follow up in treatment group	#
	Number with outcome in contro 1st follow up	State number with outcomeat 1st follow up for control group	#
	Sample size 1st follow up control	State sample size at for control group at 1st follow up	#
	Proportion with outcome in contol group 1st follow up	State proportion with outcome at 1st follow up in control group	#
Regression data	OLS	OLS used?	1=Yes 2=No
	Logistic	Logistic used?	1=Yes 2=No
	Type of logistic	What type of logistic regression?	1=binomial 2=multinomial
	GLS/WLS	GLS or WLS used?	1=Yes 2=No
	Poisson	Poisson regression used?	1=Yes 2=No
	other regression types	Other regression type used? Specify	open answer
	multilevel models	Is this a multilevel model?	1=Yes 2=No
	continous outcome	Is the outcome continous?	1=Yes 2=No
	dichotomus outcome	Is the outcome dichotomus?	1=Yes 2=No
	multiple outcome categories	Does the outcome have more than 2 categories?	1=Yes 2=No 3=Continous
	type of coefficient	What is the coefficient type?	1=raw 2=standardized 3=other
	coefficient	What is the coefficient estimate?	#
	standard error	What is the standard error of the coefficient estimate?	#
	t test	What is the t statistic associated with the focal predictor?	#

**Table:** Study design details and Risk of bias tool

	Description	Question	Coding
ID	Unique study identification #	Study	E.g. PES001
	Paper	Surname / year of first author of paper for effect size data extraction	Open answer
Research methods ‐ study design and risk of bias	Design type	What type of study design is used?	1= Randomised controlled trial (RCT) (random assignment to households/individuals) 2= Cluster‐RCT 3= RDD (quasi‐experiment with discontinuity assignment) 4 = CBA (comparison group with baseline and endline data collection) 5=Panel data, but no baseline 6 = Comparison group with endline data only 7= Natural experiment 8= Other
	Methods used for analysis	Which methods are used to control for selection bias and confounding?	1= PSM 2= Covariate matching 3= DID 4= IV‐regression 5=Heckman selection model 6= Fixed effects regression 7= Other regression 8= Randomised study
	Design and analysis method description	Briefly describe the study design and analysis method undertaken by the authors	Open answer
	Selection bias and confounding	1: Selection bias and confounding: was the identification method free from any sources of bias or were sources of bias adequately corrected for with an appropriate method of analysis?	1= Yes, 2 = No, 8 = Unclear
	Selection bias and confounding	Justification for coding decision (Include a brief summary of justification for rating, mentioning your response to all sub questions, cite relevant pages)	Open answer
	Spill‐overs, cross‐overs and contamination	2: Spill‐overs, cross‐overs and contamination: was the study adequately protected against spill‐overs, cross‐overs and contamination?	1= Yes, 2 = No, 8 = Unclear
	Spill‐overs, cross‐overs and contamination	Justification for coding decision (Include a brief summary of justification for rating, mentioning your response to all sub questions, cite relevant pages)	Open answer
	Outcome reporting	3: Outcome reporting: was the study free from selective outcome reporting?	1= Yes, 2 = No, 8 = Unclear
	Outcome reporting	Justification for coding decision (Include a brief summary of justification for rating, mentioning your response to all sub questions, cite relevant pages)	Open answer
	Analysis reporting	4: Analysis reporting: was the study free from selective analysis reporting?	1= Yes, 2 = No, 8 = Unclear
	Analysis reporting	Justification for coding decision (Include a brief summary of justification for rating, mentioning your response to all sub questions, cite relevant pages)	Open answer
	Performance bias	5: Performance bias: was the process of being observed free from motivation bias?	1= Yes, 2 = No, 8 = Unclear
	Performance bias	Justification for coding decision (Include a brief summary of justification for rating, mentioning your response to all sub questions, cite relevant pages)	Open answer
	Other bias	6: Other risks of bias: Is the study free from other sources of bias?	1= Yes, 2 = No, 8 = Unclear
	Other bias	Justification for coding decision (Include a brief summary of justification for rating, mentioning your response to all sub questions, cite relevant pages)	Open answer
	Type of comparison group		1=No intervention (business as usual) 2=Other intervention 3=Placebo control 4=Pipeline (wait‐list) control
	Other intervention differentially received by comparison group	Describe any non‐environmental comparison group intervention received which treatment group does not?	Open answer
	Unit of analysis	Are there any unit of analysis errors? (eg: the unit of analysis is different from the unit of treatement allocation and authors do not correct for these unit of analysis differences)?	1=Yes 2=No 8=Not clear 9= N/A
	Blinded participants	Blinding of participants?	1=Yes 2=No 9= N/A
	Blinded observers	Blinding of outcome assessors?	1=Yes 2=No 9= N/A
	Blinded analysts	Blinding of data analysts	1=Yes 2=No 9= N/A
	Method used to blind	Describe method(s) used to blind	Open answer (including describe method of placebo control)

**Table:** Mixed‐methods critical appraisal tool to be used for critical appraisal for qualitative studies, process evaluations and descriptive quantitative studies

**Study type**	**Methodological appraisal criteria**				**Response**		
					Yes	No	Comment
*Screening questions: assessing ‘fatal flaws’* (*Dixon‐Woods 2005* *)* *Aggregative ‘fatal flaws’ based on Stewart et al (2014)* *Configurative ‘fatal flaws’ based on Pawson (2003) TAPUS framework*	Aggregative assessment: ✓ Study reports primary data and applied methods ✓ Study reports before and after data^1^ ✓ Study features an intervention and control group						
	Configurative assessment: ✓ Study reports primary data and applied methods ✓ Study states clear research questions and objectives ✓ Study states clear research design, which is appropriate to address the stated research question and objectives (*Purposivity*) ✓ The findings of the study are based on collected data, which justify the knowledge claims (*Accuracy*)						
	***Screening question based on abstract and/or superficial reading of full‐text: Further appraisal is not feasible or appropriate when the answer is ‘No’ to any of the above screening questions!***						

**Study type**	**Methodological appraisal criteria**				**Response**		
					Yes	No	Comment / Confidence judgment
*1. Qualitative* *e.g.* *(A) Ethnography* *(B) Phenomenology* *(C) Narrative* *(D) Grounded theory* *(E) Case study* * *	**I. RESEARCH IS DEFENSIBLE IN DESIGN** (providing a research strategy that addresses the question) Appraisal indicators: ✓ *Is the research design clearly specified and appropriate for aims and objectives of the research?* Consider whether						
	*i. there is a discussion of the rationale for the study design*						
	*ii. the research question is clear, and suited to qualitative inquiry*						
	*iii. there are convincing arguments for different features of the study design*						
	*iv. limitations of the research design and implications for the research evidence are discussed*						
	**Defensible**	**Arguable**	**Critical**	**Not defensible**	*Worth to continue:*		

	**II. RESEARCH FEATURES AN APPROPRIATE SAMPLE** (following an adequate strategy for selection of participants) Appraisal indicators: Consider whether						
	*i. there is a description of study location and how/why it was chosen*						
	*ii. the researcher has explained how the participants were selected*						
	*iii. the selected participants were appropriate to collect rich and relevant data*						
	*iv. reasons are given why potential participants chose not take part in study*						
	**Appropriate sample**	**Functional sample**	**Critical sample**	**Flawed sample**	*Worth to continue:*		

	**III. RESEARCH IS RIGOROUS IN CONDUCT** (providing a systematic and transparent account of the research process) Appraisal indicators: Consider whether						
	*i. researchers provide a clear account/description of the process by which data was collected (e.g. for interview method, is there an indication of how interviews were conducted?/procedures for collection or recording of data?)*						
	*ii. researchers demonstrate that data collection targeted depth, detail and richness of information (e.g. interview/observation schedule)*						
	*iii. there is evidence of how descriptive analytical categories, classes, labels, etc. have been generated and used*						
	*iv. presentation of data distinguishes clearly between the data, the analytical frame used, and the interpretation*						
	*v. methods were modified during the study; and if so, has the researcher explained how and why?*						
	**Rigorous conduct**	**Considerate conduct**	**Critical conduct**	**Flawed conduct**	*Worth to continue:*		

	**IV. RESEARCH FINDINGS ARE CREDIBLE IN CLAIM/BASED ON DATA** (providing well‐founded and plausible arguments based on the evidence generated) Appraisal indicators: Consider whether						
	*i. there is a clear description of the form of the original data*						
	*ii. sufficient amount of data are presented to support interpretations and findings/conclusions*						
	*iii. the researchers explain how the data presented were selected from the original sample to feed into the analysis process (i.e. commentary and cited data relate; there is an analytical context to cited data, not simply repeated description; is there an account of frequency of presented data?)*						
	*iv. there is a clear and transparent link between data, interpretation, and findings/conclusion*						
	*v. there is evidence (of attempts) to give attention to negative cases/outliers etc.*						
	**Credible claims**	**Arguable claims**	**Doubtful claims**	**Not credible**	*If findings not credible, can data still be used?*		

	**V. REASEARCH ATTENDS TO CONTEXTS** (describing the contexts and particulars of the study) Appraisal indicators: Consider whether						
	*i. there is an adequate description of the contexts of data sources and how they are retained and portrayed?*						
	*ii. participants' perspectives/observations are placed in personal contexts*						
	*iii. appropriate consideration is given to how findings relate to the contexts (how findings are influenced by or influence the context)*						
	*iv. the study makes any claims (implicit or explicit) that infer generalisation (if yes, comment on appropriateness)*						
	**Context central**	**Context considered**	**Context mentioned**	**No context attention**			

	**VI. RESEARCH IS REFLECTIVE** (assessing what factors might have shaped the form and output of research) Appraisal indicators: Consider whether						
	*i. appropriate consideration is given to how findings relate to researchers' influence/own role during analysis and selection of data for presentation*						
	*ii. researchers have attempted to validate the credibility of findings (e.g. triangulation, respondent validation, more than one analyst)*						
	*iii. researchers explain their reaction to critical events that occurred during the study*						
	*iv. researchers discuss ideological perspectives/values/philosophies and their impact on the methodological or other substantive content of the research (implicit/explicit)*						
	**Reflection**	**Consideration**	**Acknowledgement**	**Unreflective research**	*NB: Can override previous exclusion!*		
**OVERALL DECISCON – EXLUDE / INCLUDE** (study generates new knowledge relevant to the review question and complies with minimum criteria to ensure reliability and empirical grounding of knowledge)							

Sources used in this section (in alphabetical order); Campbell et al (2003); CASP (2006); CRD (2009); Dixon‐Woods et al (2004); Dixon‐Woods et al (2006)^cited in Gough 2012^; Greenhalgh & Brown (2014); Harden et al (2004)^cited in SCIE & Gough 2012^; Harden et al (2009); Harden & Gough (2012); Mays & Pope (1995); Pluye et al (2011); Spencer et al 2006; Thomas et al (2003); SCIE (2010).							

**Study type**	**Methodological appraisal criteria**				**Response**		
					Yes	No	Comment / risk of bias judgment
*2. Quantitative* *(non‐randomised; Randomised‐Controlled)* *Common non‐random design include:* *(A) Non‐randomised CT* *(B) Cohort studies* *(C) Case‐control* *(D) Cross‐sectional analytical studies* *Most common ways of controlling for bias due to baseline confounding:* *• Matching attempts to emulate randomization* *• Propensity score matching and methods* *• Stratification where sub‐groups have been compared* *• Regression analysis where covariates are adjusted for* *Randomised designs: Randomised Control Trial (RCT)*	I. **Selection bias:** (Are participants recruited in a way that minimizes selection bias?) Appraisal indicators: Consider whether						
	*i. there is a clear description of how and why sample was chosen*						
	*ii. there is adequate sample size to allow for representative and/or statistically significant conclusions*						
	*iii. participants recruited in the control group were sampled from the same population as that of the treatment*						
	*iv. group allocation process attempted to control for potential risk of bias*						
	**Low risk of bias**	**Risk of bias**	**High risk of bias**	**Critical risk of bias**	*Worth to continue:*		

	**II. Bias due to baseline confounding:** (Is confounding potentially controllable in the context of this study?) Appraisal indicators: Consider whether						
	*i. the treatment and control group are comparable at baseline*						
	*ii. matching was applied, and in case, featured sufficient criteria*						
	*iii. the authors conducted an appropriate analysis that controlled for all potential critical confounding domains*						
	iv. *the authors avoided to adjust for post‐intervention variables*						
	**Low risk of bias**	**Risk of bias**	**High risk of bias**	**Critical risk of bias**	*Worth to continue:*		

	**IF RANDOMISED CONTROL TRIAL, SKIP I + II AND START HERE!** **Bias due to ineffective randomisation**: (Is allocation of treatment status truly random?) Appraisal indicators: Consider whether						
	*i. there is a clear description of the randomisation process*						
	*ii. the unit of randomisation and number of participants is clearly stated (pay special attention to treatment and control locations/ balance)*						
	*iii. eligibility criteria for study entry are specified*						
	*iv. characteristics of baseline and endline sample are provided* ^1^						*Preferable condition, see 1*
	**Low risk of bias**	**Risk of bias**	**High risk of bias**	**Critical risk of bias**	*If critical risk of bias, treat as non‐random study*		

	**III. Bias due to departures from intended interventions** (Was the intervention implemented as laid out in the study protocol?) Appraisal indicators: Consider whether						
	*i. the critical co‐interventions were balanced across intervention groups*						
	*ii. treatment switches were low enough to not threaten the validity of the estimated effect of intervention*						
	*iii. implementation failure was minor and unlikely to threaten the validity of the outcome estimate*						
	*iv. it is possible that intervention was taken by the controls (contamination and possible crossing‐over)**						**whilst challenging in terms of estimating impact, spill‐overs might be an important finding in itself (eg teachers read to pupils/village/family members)*
	*v. it is possible that knowledge of the intervention group affects how the two study groups are treated in course of follow‐up by investigators?***						***consider only in extreme cases in which preferential treatment is clearly evident; blinding in general not expected in social interventions*
	**Low risk of bias**	**Risk of bias**	**High risk of bias**	**Critical risk of bias**	*Worth to continue:*		

	**IV. Bias due to missing data (attrition)** (Are the intervention groups free of critical differences in participants with missing data?) Appraisal indicators: Consider whether						
	*i. outcome data are reasonably complete (80% or above)*						
	*ii. If ‘no’, are missing data reported?*						
	*iii. If missing data:are proportion of participants and reasons for missing data similar across groups?*						
	*iv. If missing data: Were appropriate statistical methods used to account for missing data? (e.g. sensitivity analysis)*						
	*v. If not possible to control for missing data, are outcomes with missing data excluded from analysis?*						
	**Low risk of bias**	**Risk of bias**	**High risk of bias**	**Critical risk of bias**	*Worth to continue:*		

	**V. Outcome reporting bias** (Are measurements appropriate, e.g. clear origin, or validity known?) Appraisal indicators: Consider whether						
	*i. there was an adequate period for follow up****						****in many social science interventions, follow‐up is not required to coincide with the start of the treatment; further, longer period of follow up are often required to measure changes. In the context of education, the question of retention – in particular when dealing with short intervention periods –(&lt; 1 month) is of major interest.*
	*ii. the outcome measure was clearly defined and objective*						
	*iii. outcomes were assessed using standardised instruments and indicators*						
	*iv. outcome measurements reflect what the experiment set out to measure*						
	*v. the methods of outcome assessment were comparable across experiential groups*						
	**Low risk of bias**	**Risk of bias**	**High risk of bias**	**Critical risk of bias**	*Worth to continue:*		

	**VI. Bias in selection of results reported** (Are the reported outcomes consistent with the proposed outcomes at the protocol stage?) Appraisal indicators: Consider whether						
	*i. it is unlikely that the reported effect estimate is available primarily because it was a notable finding among numerous exploratory analyses*						
	*ii. it is unlikely that the reported effect estimate is prone to selective reporting from among multiple outcome measurements within the outcome domain*						
	*iii. it is unlikely that the reported effect estimate is prone to selective reporting from among multiple analyses of the outcome measurements*						
	*iv. the analysis includes an intention to treat analysis? (If so, was this appropriate and were appropriate methods used to account for missing data?)*****						*****usually in clinical RCTs, rare in social science: only rate if conducted*
	**Low risk of bias**	**Risk of bias**	**High risk of bias**	**Critical risk of bias**			
**OVERAL RISK OF BIAS:**							

Sources used in this section (in weighted order): Cochrane (2014); Stewart et al (2014); Stewart et al (2012); Higgins et al (2011); Greenhalgh & Brown (2014); Pluye et al (2011); Gough et al (2007)							

**Study type**	**Methodological appraisal criteria**				**Response**		
					Yes	No	Comment /confidence judgment
*3. Mixed‐methods* ^2^ *Sequential explanatory design* *The quantitative component is followed by the qualitative. The purpose is to explain quantitative results using qualitative findings. E.g., the quantitative results guide the selection of qualitative data sources and data collection, and the qualitative findings contribute to the interpretation of quantitative results.* *Sequential exploratory design* *The qualitative component is followed by the quantitative. The purpose is to explore, develop and test an instrument (or taxonomy), or a conceptual framework (or theoretical model). E.g., the qualitative findings inform the quantitative data collection, and the quantitative results allow a generalization of the qualitative findings.* *Triangulation designs* *The qualitative and quantitative components are concomitant. The purpose is to examine the same phenomenon by interpreting qualitative and quantitative results (bringing data analysis together at the interpretation stage), or by integrating qualitative and quantitative datasets (e.g., data on same cases), or by transforming data (e.g., quantization of qualitative data).* *Embedded/convergent design* *The qualitative and quantitative components are concomitant. The purpose is to support a qualitative study with a quantitative sub‐study (measures), or to better understand a specific issue of a quantitative study using a qualitative sub‐study, e.g., the efficacy or the implementation of an intervention based on the views of participants.*	**I. RESEARCH INTEGRATION/SYNTHESIS OF METHODS** (assessing the value‐added of the mixed‐methods approach) Applied mixed‐methods design: ○ Sequential explanatory design ○ Sequential explorative design ○ Triangulation design ○ Embedded design Appraisal indicators: Consider whether						
	*i. the rationale for integrating qualitative and quantitative methods to answer the research question is explained* *[DEFENSIBLE]*						
	*ii. the mixed‐methods research design is relevant to address the qualitative and quantitative research questions, or the qualitative and quantitative aspects of the mixed methods research question* *[DEFENSIBLE]*						
	*iii. there is evidence that data gathered by both research methods was brought together to inform new findings to answer the mixed‐methods research question (e.g. form a complete picture, synthesise findings, configuration)* *[CREDIBLE]*						
	*iv. the approach to data integration is transparent and rigorous in considering all findings from both the qualitative and quantitative module (danger of cherry‐picking)* *[RIGOROUS]*						
	*v. appropriate consideration is given to the limitations associated with this integration, e.g., the divergence of qualitative and quantitative data (or results)?* *[REFLEXIVE]*						
For mixed‐methods research studies, each component undergoes its individual critical appraisal first. Since qualitative studies are either included or excluded, no combined risk of bias assessment is facilitated, and the assigned risk of bias from the quantitative component similarly holds for the mixed‐methods research. The above appraisal indicators only refer to the applied mixed‐methods design. If this design is not found to comply with each of the four mixed‐methods appraisal criteria below, then the quantitative/qualitative components will individually be included in the review:							
Mixed‐methods critical appraisal: 1. Research is defensible in design 2. Research is rigorous in conduct 3. Research is credible in claim 4. Research is reflective	Qualitative critical appraisal: Include / Exclude				Quantitative critical appraisal: 1. Low risk of bias 2. Risk of bias 3. High risk of bias 4. Critical risk of bias		
Combined appraisal: Include / Exclude mixed‐methods findings judged with____________________________ risk of bias							

Section based on Pluye et al (2011). Further sources consulted (in alphabetical order): Creswell & Clark (2007); Crow (2013); Long (2005); O'Cathain et al (2008); O'Cathain (2010); Pluye & Hong (2014); Sirriyeh et al (2011).							

1 Two theoretical exceptions to this rule apply:
i) A RCT with appropriate randomization procedure can be included without showing baseline data, as both experimental groups can be assumed to be equal at baseline by design. ii) A sophisticated quasi‐experimental design such as PSM or RDD in theory could make the same claim to not require baseline data.
In both cases, the advise of an evaluation specialist will be thought as the researcher does not have the capacity to make an informed judgment in such specialist cases.

2 The mixed‐methods Critical Appraisal is facilitated for studies applying an explicit mixed‐methods approach. The component is applied in addition to criteria for the qualitative component (I to VI), and appropriate criteria for the quantitative component (I to VI).
